# Metal–Organic Frameworks and Their Biodegradable Composites for Controlled Delivery of Antimicrobial Drugs

**DOI:** 10.3390/pharmaceutics15010274

**Published:** 2023-01-12

**Authors:** Tayah C. Livesey, Lila A. M. Mahmoud, Maria G. Katsikogianni, Sanjit Nayak

**Affiliations:** 1 School of Chemistry and Biosciences, University of Bradford, Bradford BD7 1DP, UK; 2 School of Pharmacy, Al-Zaytoonah University of Jordan, Amman 11733, Jordan

**Keywords:** MOFs, antimicrobial resistance (AMR), polymers, drug delivery, composite, biodegradable

## Abstract

Antimicrobial resistance (AMR) is a growing global crisis with an increasing number of untreatable or exceedingly difficult-to-treat bacterial infections, due to their growing resistance to existing drugs. It is predicted that AMR will be the leading cause of death by 2050. In addition to ongoing efforts on preventive strategies and infection control, there is ongoing research towards the development of novel vaccines, antimicrobial agents, and optimised diagnostic practices to address AMR. However, developing new therapeutic agents and medicines can be a lengthy process. Therefore, there is a parallel ongoing worldwide effort to develop materials for optimised drug delivery to improve efficacy and minimise AMR. Examples of such materials include functionalisation of surfaces so that they can become self-disinfecting or non-fouling, and the development of nanoparticles with promising antimicrobial properties attributed to their ability to damage numerous essential components of pathogens. A relatively new class of materials, metal–organic frameworks (MOFs), is also being investigated for their ability to act as carriers of antimicrobial agents, because of their ultrahigh porosity and modular structures, which can be engineered to control the delivery mechanism of loaded drugs. Biodegradable polymers have also been found to show promising applications as antimicrobial carriers; and, recently, several studies have been reported on delivery of antimicrobial drugs using composites of MOF and biodegradable polymers. This review article reflects on MOFs and polymer–MOF composites, as carriers and delivery agents of antimicrobial drugs, that have been studied recently, and provides an overview of the state of the art in this highly topical area of research.

## 1. Introduction

Pathogens, such as viruses, bacteria, and fungi, can lead to the development of various diseases [1]. Pathogens are contagious and, through evolution, they can become resistant to innate immunity, therefore requiring medical intervention to be eradicated [2]. Antimicrobials, in particular antibiotics, are used to treat infections caused by these pathogens in everyday health care and in clinical therapy. They are also used preventatively in surgical procedures, organ transplants, cancer treatments, and wound healing. Additionally, they are widely used in agriculture, farming, and the food industry [3,4].

These antimicrobials tend to work by either decreasing, blocking, or stopping the growth of pathogens [5]. They also act by targeting several vital cell functions, which include the biological synthesis of their plasma membranes, nucleic acids, and proteins. This results in the inhibition of these processes and thus significantly reduces the chances of pathogen survival [6]. The mechanism of action of these antimicrobials is tailored to act either against Gram-positive or Gram-negative bacteria. However, some antimicrobial drugs treat both Gram-positive and Gram-negative infections, and are classed as broad-spectrum antibiotics [7].

With medical intervention, such as upon using antibiotics, microbes can develop a resistance to antimicrobial drugs, rendering them ineffective, which is known as antimicrobial resistance (AMR) [8,9]. AMR is an adaptive mechanism that provides selection pressure from external agents [10]. Thus, to keep up with these adaptive changes, there is a continuous battle between the development of alternative treatments and these microbes becoming resistant to antimicrobials. Multidrug-resistant pathogens have an AMR to a minimum of three antimicrobial agents. There are some microbes, such as some *Mycobacterium tuberculosis* strains, that have been found to be resistant to nearly all available forms of antimicrobials [11]. It is predicted that by 2050, antimicrobial-resistant infections will become the most predominant cause of death globally, leading to more than 10 million lives being taken annually, if this problem is not tackled [11,12,13]. There are multiple strategies which can be implemented to aid in reducing AMR. One method is through the introduction of good personal hygiene, good practice, infection control and precautions, such as practicing antimicrobial stewardship, through appropriate prescription of antibiotics in clinical settings. In addition to these ongoing efforts of preventive strategies and infection control, there is continuous ongoing research on the development of novel vaccines, antimicrobial agents, and optimised diagnostic tools to address AMR [9], along with parallel efforts to develop materials for optimised drug delivery. Examples of such materials include surface-functionalised materials that can become self-disinfecting or non-fouling, and the development of nanoparticles with promising antimicrobial properties [14,15]. Metal–organic frameworks (MOFs) are a relatively new class of porous materials which have caught great attention in this area and are being investigated for their ability to act as carriers of antimicrobial agents, because of their ultrahigh porosity and modular structures which can be engineered to control the delivery mechanism of loaded drugs [16]. Biodegradable polymers have also been found to show promising applications as antimicrobial carriers [17,18]. Recently, several studies have been reported on delivery of antimicrobial drugs using composites of these two classes of materials. This review article reflects on recently reported MOFs and biodegradable polymer–MOF composites as carrier and delivery agents for antimicrobial drugs and provides an overview of the state of the art in this highly topical area of research.

## 2. Metal–Organic Frameworks (MOFs)

**Figure 1:**
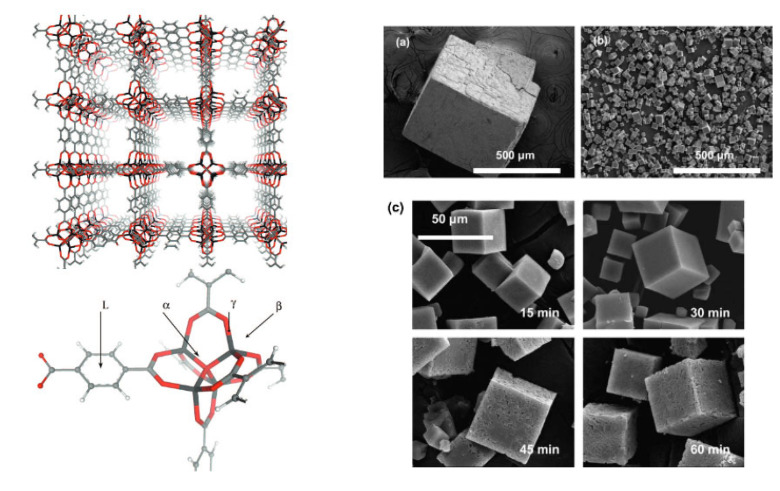
(**Left**): Crystallographic view of MOF-5; (**Right**): SEM images of MOF-5 crystals synthesised using (**a**) solvothermal method, (**b**) microwave, and (**c**) degradation of MOF crystals as a result of microwave irradiation for 15, 30, 45, and 60 min. Reproduced with permission from [32,35].

Metal–organic frameworks are a class of compounds that consist of inorganic and organic secondary building units (SBUs) bonded together to form a three-dimensional extended lattice with potential voids [19,20]. MOFs have attracted great attention since they were first reported, approximately two decades ago, particularly due to their porous structures and ultrahigh surface area, which makes them attractive for various applications, such as gas storage, separation, catalysis, and energy storage [21,22,23,24,25,26,27,28,29,30,31]. The SBUs of MOFs are formed of metal ions or polymetallic cluster nodes and organic linkers, sometimes called “struts”. The directional properties of the coordination bonds of metal ions and cluster nodes as well as the geometry of the organic linkers allow for the formation of highly ordered three-dimensional networks, with regular channels and pores. Figure 1 shows the porous feature of one of the first-reported MOFs, MOF-5 [32]. Such a lattice arrangement often results in highly ordered crystalline materials, as shown from Scanning Electron Microscopy (SEM) images of cubic MOF-5 crystals (Figure 1). Many methods of synthesis of MOFs have been reported, such as solvothermal methods, microwave-assisted synthesis, and mechanochemical methods [33]. It is also worth noting that defects in these crystalline materials can sometimes be very useful for certain applications, including drug delivery [33]. Variations in reaction conditions can be used to strategically induce some disorder in these lattice arrangements, causing defects [34].

**Figure 2:**
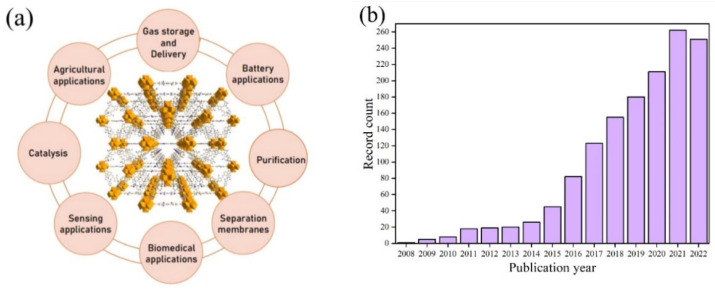
(**a**) Scheme showing a range of potential applications of MOFs. MOF image reproduced with permission from [46] (**b**) A graphical representation of the record count per year of publication, from a literature search on Web of Science (as in December 2022) for “MOFs” and “drug delivery”, indicating the rapid growth in interest in this area of research.

MOFs can be designed with a variety of different geometries, sizes, and functionalities. The organic linkers are generally made up of polytopic carboxylates or other ligand systems, such as imidazoles [36]. Once bonded to the metallic nodes, an open porous framework is formed. The porosity of MOFs often makes up >50% of the crystals volume with large surface areas with a theoretical limit of 10,436 m^2^g^−1^ [37]. Structures with a “permanent porosity” tend to be available in a wide range of forms. These characteristics, along with their possible post-synthetic modifications (PSMs) to incorporate numerous functional groups, make MOFs promising for a wide variety of applications, such as hydrogen storage, carbon capture, catalysis, separation, battery applications, water treatment, and agrochemical delivery (Figure 2a) [38,39,40,41]. Research has also shown that due to their microporous structures, MOFs can host drug molecules and act as a delivery vehicle for medicinal and imaging compounds. For example, they can be used to deliver anticancer drugs and cancer markers [42,43,44,45]. High porosity of the MOFs allows them to have good drug uptake, controlled release and sustained delivery. There are multiple factors that influence the ability of a MOF to act as an effective carrier system for drugs. For example, porosity, pore dimensions, surface area, and functional groups on linkers of the MOFs are all important parameters that have a direct influence over an effective interaction between a MOF and a drug molecule, and hence influence the loading capacity and the delivery mechanism. Due to their high loading capacity and customisable functional structures, MOFs are gaining increasing interest for drug delivery applications, and the field has evolved exponentially since the first study was reported, as can be seen from the rapidly increasing number of articles published on MOFs for drug delivery applications (Figure 2b).

MOFs can act as delivery vehicles for antimicrobial agents in two different ways: (1) Due to the antimicrobial effects of the constituents of the MOFs. In this pathway, either the metal ions or the ligands (or both) can act as antimicrobial agents upon slow decomposition of the MOFs [47]. (2) Due to loaded antimicrobial drugs. This pathway takes advantage of the microporous structure of the MOFs, and the desired drugs can be loaded and then released in a controlled rate over a long period, either by slow diffusion of the drugs or by decomposition of the MOFs or a combination of the two [48,49,50]. This is an advantage over alternative drug carrier systems, such as nanocarriers, which have the tendency of releasing the drugs in an uncontrolled manner, by burst release [51,52]. The release mechanism depends on several factors, such as the stability of the MOFs, the pore dimensions, and the interactions between the drugs and MOFs. Based on the properties of the MOFs, they can be loaded with drugs which have hydrophilic, hydrophobic, and amphiphilic character. The MOFs can also carry some loaded metal ions, such as Ag^+^, that have antimicrobial properties and can be delivered in a controlled manner.

**Figure 3:**
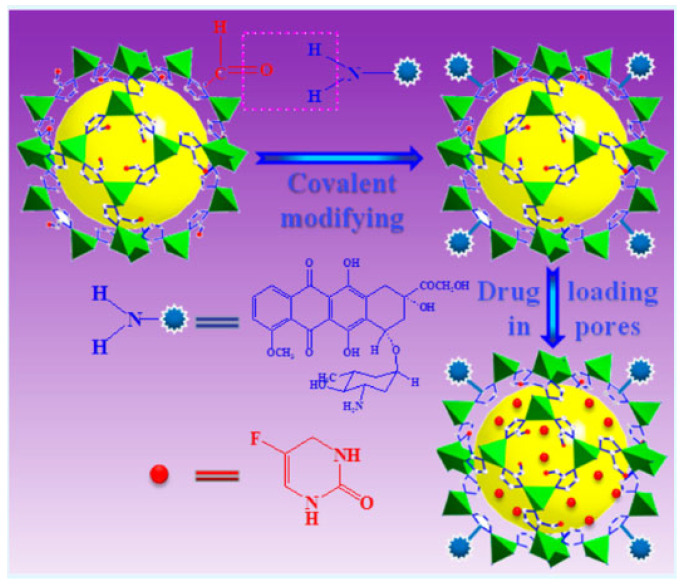
Two mechanisms through which drugs can be loaded into MOFs are shown. Covalently bonded doxorubicin (Blue), and non-covalently loaded 5-floururacil (red) inside MOF pores of ZIF-90. Reproduced with permission from [53].

**Figure 4:**
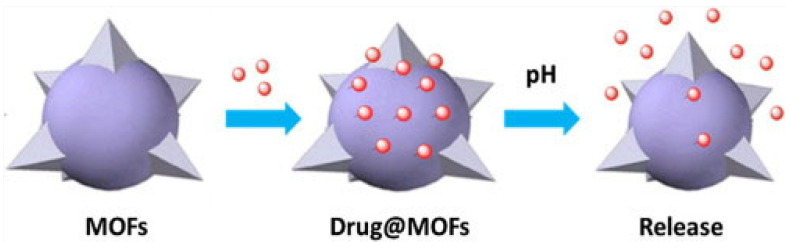
This image displays uptake and then release of a drug from MOFs triggered by a change in pH. Reproduced with permission from [55].

The drugs can be loaded into MOFs, either by covalent or non-covalent interactions (Figure 3) [53]. Several strategies have been used to increase the efficiency of drug loading [33,54]. One such approach is the introduction of defects in the MOF structure, to create unsaturated coordination sites or larger empty spaces inside the MOFs [55]. Additionally, several external stimuli, such as pH, can be used to aid delivery of the loaded drugs at targeted sites (Figure 4) [55].

## 3. MOFs as Antimicrobial Agents

**Figure 5:**
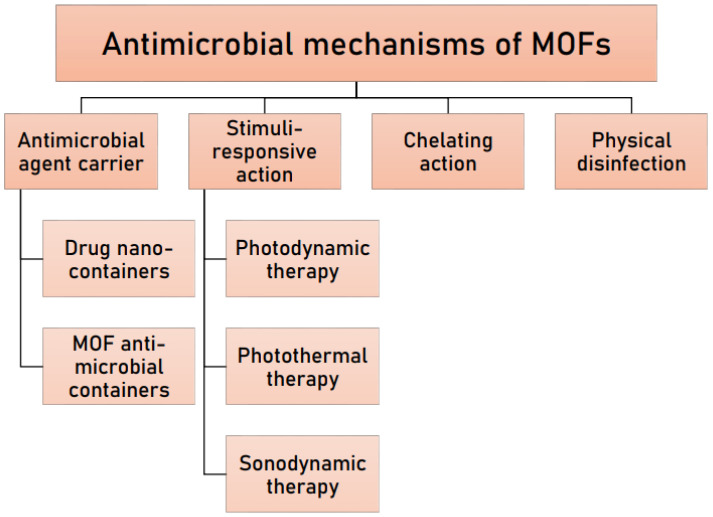
A scheme showing the different mechanisms of the antimicrobial action of MOFs that are discussed in this review.

In this section, various mechanisms of the antimicrobial action of MOFs are discussed (Figure 5). In general, some of these mechanisms can be due to structural attributes in the MOF framework, as well as a result of their further functionalisation and modification. As will be shown below, the antimicrobial action of a MOF can be due to (1) slow release of loaded antimicrobial agents, by acting as a reservoir for the extended release of antimicrobial molecules which are often held inside the MOFs by supramolecular forces; (2) degradation of bioactive MOFs and release of effective metal ions and/or linkers; (3) acting as a chelating agent; (4) photoactivity due to the presence of photosensitiser molecules; and (5) through physical disinfection. As will be shown by some of the studies below, sometimes the overall antimicrobial effect can be a combination of more than one of these mechanisms, providing a synergistic antimicrobial effect. A summary of recent studies on the use of MOFs for delivering antimicrobial drugs is presented in Table 1.

In the following sections, various mechanisms will be discussed.

### 3.1. MOFs as Antimicrobial Agent Carrier

**Figure 6:**
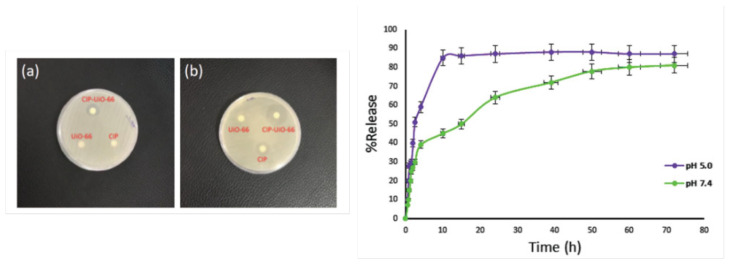
(**Left**): Disk diffusion studies on (**a**) *S. aureus* and (**b**) *E. coli.* (**Right**): pH-stimulated release of CIP from UiO-66. Reproduced with permission from [60].

The multifaceted nature of MOFs allows them to be excellent candidates for antimicrobial applications. Due to their high porosity and functional nature, MOFs have shown high loading capacities (Table 1), when used as a vehicle for antimicrobial drug delivery platform. In general, drugs are loaded inside the MOFs post-synthetically. Pre-synthesised MOFs are stirred in a drug solution, and then filtered out. This method has some advantages as the amount of drug loading can be controlled, by changing factors such as concentration and time of stirring. However, some MOF structures can disintegrate during post-synthetic drug loading [56]. Another method of drug loading can be in situ during synthesis of the MOFs [57,58,59]. For example, in a study by Nasrabadi et al., a high loading capacity of 84 wt% was found for ciprofloxacin (CIP) in a Zr-based MOF; UiO-66, as nano-containers for the slow release of the drug [60]. UiO-66 was synthesised solvothermally, and was post-synthetically loaded with CIP by stirring the MOF/CIP suspension for five days. Release studies have demonstrated pH-sensitive release, owing to its slow degradation behaviour in acidic conditions. Disk diffusion studies showed an increased antimicrobial effect against *Escherichia (E.) coli* and *Staphylococcus* (*S.*) *aureus.* For CIP-loaded UiO-66, inhibition zones of 22 and 24 mm were observed for *E. coli* and *S. aureus*, respectively, compared to 14 mm and zero when CIP was used alone, as shown in Figure 6. The increased efficiency was attributed to possible combined antimicrobial effect of the UiO-66 components and the released drug.

**Figure 7:**
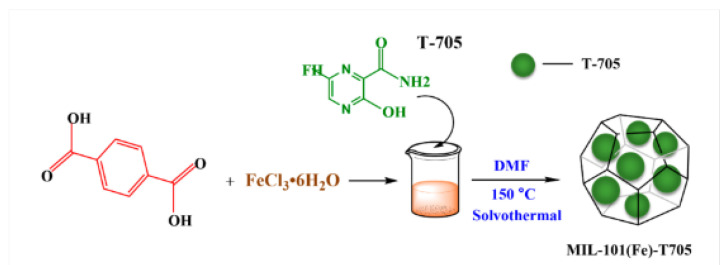
Scheme showing loading of favipiravir in MIL-101(Fe) during in situ synthesis. Reproduced with permission from [69].

The antimicrobial effect of loaded MOFs relies on its slow and extended release of the drug. Extended-release formulations have been proven to be effective at maximising the therapeutic effect of antimicrobials [61], by maintaining the drug concentration above the minimum inhibitory concentration (MIC) for a prolonged period. Some antibiotic drug classes such as *β*-lactams, tetracyclines, and cephalosporins have a time-dependent therapeutic effect [62]. This means that the MIC is a threshold for the antimicrobial action [63], and that its efficacy relies on the time of exposure, at concentrations above the MIC, rather than the concentration itself. In addition, prolonged exposure to concentrations below this threshold can promote the development of AMR strains [64], requiring a prolonged maintenance of this concentration, via a slow-releasing drug carrier. In 2019, Gallis et al. incorporated the cephalosporin antibiotic, ceftazidime, inside the pores of ZIF-8 [65]. ZIF-8 was loaded post-synthetically, by stirring in an aqueous solution of the drug for three days, reaching a maximum loading capacity of 10.9 wt%. Release studies have shown an extended release of up to a week, in addition to showing antimicrobial action against *E. coli*. Metal ions such as Ag^+^ are well known to possess antimicrobial properties [66]. In 2022, Li et al. used the iron-based MOF MIL-101(Fe), as a carrier for loading silver nanoparticles, showing a lower MIC value than using MIL-101(Fe) alone [67]. Thus, proving that the antimicrobial effect can be mainly attributed to the release of Ag^+^ from the MOF pores. Another study has explored the slow release of Ag^+^ ions from postmetalated two Zr-based MOFs, UiO-66−2COOH and UiO-67-bpydc [68]. in this study, Mortada et al. have post-synthetically modified the MOFs with silver cations that can bind to the organic linker, thus providing a reservoir of slowly released silver. Their efficacy as an antimicrobial agent was assessed against *E*. *coli*. A minimum bactericidal concentration (MBC) of as low as 6.5 µg mL^−1^ of silver content was found. It was also shown that the growth of these bacteria was completely inhibited at concentrations as low as 175 ng mL^−1^ (MIC). Another study in 2022 used MIL-101(Fe) MOF for slow release of the antiviral agent, favipiravir (T705) [69]. Favipiravir was loaded in situ and different ratios of favipiravir were explored for optimised loading (Figure 7).

The Braeuer–Emmett–Teller (BET) surface area shows a reduction from 199.7 to 116.8 m^2^g^−1^ upon loading of the pristine MIL-101(Fe) with favipiravir. The drug content of loaded MIL-101(Fe) was determined to be 27.03% by weight, and release studies have shown the pH-dependent release, with enhanced release in acidic conditions.

**Figure 8:**
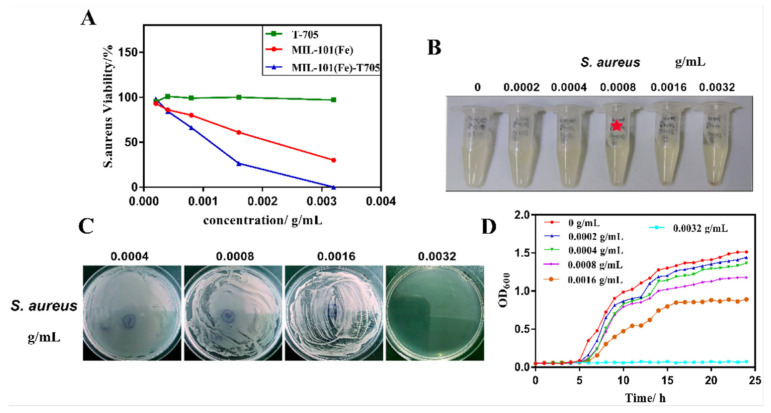
Antimicrobial studies against *S. aureus* using T705, MIL-101(Fe) and T705-loaded MIL-101(Fe). (**A**) Plot showing the viability studies on *S. aureus* of favipiravir, MIL-101(Fe) and T705-loaded MIL-101(Fe). (**B**) MIC (marked with red) using different concentrations of T705-loaded MIL-101(Fe). (**C**) Disk diffusion studies, showing MBC at 0.0032 g mL^−1^. (**D**) Growth curves under different concentrations of loaded MOFs. Reproduced with permission from [69].

As shown in Figure 8, favipiravir alone did not show any inhibitory effect against *S. aureus*. However, MIL-101(Fe) and favipiravir-loaded MIL-101(Fe) showed an inhibitory effect, with the latter being more effective, as it is shown in Figure 8A. The antimicrobial effect for favipiravir-loaded MIL-101(Fe) was proposed to be concentration dependent, with an MIC of 0.0008 g mL^−1^ and an MBC of 0.0032 g mL^−1^.

As shown in previous studies, in addition to being a reservoir for the extended release of drug cargo, the incorporation of the drug inside the MOFs can lower the MIC needed for effective killing of microbes. Another example of this can be found in a study, where Zn_2_(bdc)_2_(dabco) MOF was synthesised and loaded with gentamicin [70]. Similar powder X-ray diffraction (PXRD) patterns of the drug-loaded MOF to the pristine phase indicated maintenance of crystallinity and structural integrity. BET surface area reduced from 1256 to 1.16 m^2^g^−1^ upon loading of the drug, in addition to decrease in pore volume, indicating the drug occupying the pores. Inhibition zones indicate a better antimicrobial effect of the gentamicin loaded MOF than gentamicin alone, with a lower amount of drug needed to inhibit microbial growth. The drug release behaviour showed pH-dependence, as the pore sizes of the MOF structure increases at acidic pH, leading to increased drug release. This enhanced efficacy of loaded MOFs, compared to the conventional drug, can be explained as sometime the components of the MOF itself also possess antimicrobial properties, such as some metals including Ag^+^ and Cu^2+^, and linkers such as imidazoles. MOFs possessing such effective moieties can slowly disintegrate, and hence slowly release their antimicrobial building blocks. This was observed in a study by Taheri et al., [71]. ZIF-8 provided a reservoir for the slow release of Zn^2+^ ions, as well as 2-methyl imidazolate molecules. Antibacterial studies showed a significantly larger inhibition zone compared to ZnO, with 20.1 mm for ZIF-8 and 14.1 mm for ZnO. The MBC also showed a much better efficacy, with 250 mgL^−1^ for ZIF-8 and 600 mgL^−1^ for ZnO. In another study by Soltani et al., it was observed that gentamicin-loaded ZIF-8 shows significantly higher antimicrobial effect, with a zone of inhibition of 12 and 14 mm against *S. aureus* and *E. coli* alone [72]. The stability of MOFs can be correlated to the strength of its coordinate bonds and can be sometimes predicted by Pearson’s hard-soft acid-base (HSAB) principle [73]. The operating environment will affect the stability of coordination bonds, and hence make the framework susceptible to factors such as pH and temperature, resulting in the degradation of its components, allowing for their slow, trigger-responsive release.

**Figure 9:**
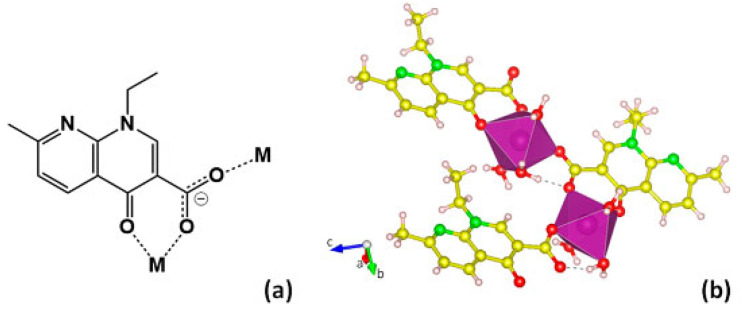
(**a**) Coordination modes of nalidixic acid in the MOF is shown (**b**) The octahedral geometry of the coordination spheres of metal centres are shown. Reproduced with permission from [74].

Antimicrobial agents can also be used as a component of MOF itself, as a linker or the metal ion. For example, in 2019, nalidixic acid, a first-generation quinolone antibacterial agent, was incorporated as the linker in Mn- and Mg-based BioMOFs (Figure 9) [74]. Both BioMOFs were synthesised mechanochemically, an alternative method of MOF synthesis that relies on mixing the metal salts and the linkers by ball milling or other mechanical processes [75]. Antimicrobial studies of the resulting BioMOFs have shown enhanced efficacy compared to nalidixic acid alone, showing effective growth inhibition of Gram-positive and Gram-negative bacteria, as well as yeasts. This increase in activity can be observed in small MIC values required for inhibition of *S. aureus* at 31.1 µg/mL for both BioMOFs, compared to at 125 µg mL^−1^ for nalidixic acid. The BioMOFs also exhibited enhanced water solubility, compared to nalidixic acid, which has low bioavailability because of its poor solubility in water.

The overall antimicrobial action of the drug-loaded MOFs is an additive effect of the drug, and the MOF components, such as the metal moiety and the ligand. In a study by Bhardwaj et al., three Zn-based MOFs, IR-MOF-3, MOF-5 and Zn-BTC, were loaded with kanamycin and ampicillin [76]. The enhanced efficacy provided an example of the synergistic and additive effects of MOF components, as well as the loaded drug cargo. 

The unloaded MOFs, kanamycin, and ampicillin and their combinations were tested against *E. coli*, *S. aureus*, *S. lentus* and *L. Monocytogenes.* The antimicrobial studies showed greater efficacy of the drug-loaded MOFs, compared to the MOF or the drug alone. This was evident by lower MIC. For example, for *S. aureus*, the MIC of ampicillin was reduced by 2-folds when loaded into Zn-BTC and MOF-5, with an MIC of 16 µg mL^−1^ compared to MOFs and ampicillin alone, with an MIC of 200 and 32 µg mL^−1^, respectively. Fractional inhibition concentrations (FIC) were determined via checkerboard microtiter tests. The FIC is usually obtained to give us an idea of the effects of individual drug components on the MIC, and in this study, the FIC indices confirmed the synergistic and additive effects of the MOF components on the antimicrobial action. Kanamycin-loaded IRMOF-3 showed a synergistic effect against *E. coli*, by having an FIC index ≤ 0.5, while other MOF–drug combinations, such as MOF-5/kanamycin, exhibited additive effect on all four microorganisms by having an FIC index of 0.75.

### 3.2. MOFs for Photoactive Antimicrobial Action

Another mechanism of the antimicrobial action of MOFs involves the use of photosensitiser compounds. Photosensitiser molecules absorb electromagnetic radiation and transfer it to its neighbouring molecules. This ability is facilitated by the presence of π-conjugated system, allowing for delocalised electrons to be photoexcited and escape the HOMO to a LUMO. This electron from the HOMO gets promoted to an excited triplet state. The photosensitiser facilitates the transfer of energy from ground-state triplet oxygen (^3^O_2_) to an excited state singlet (^1^O_2_) [77]. Singlet oxygen is a reactive oxygen species (ROS) which is known to cause oxidative damage to living cells [78], and hence have an antimicrobial effect on bacteria, fungus and other microbes, resulting photothermal lysis. Photosensitiser molecules have been widely used in photodynamic therapy (PDT), which is used to kill microbes [79]. The photosensitiser molecules can be incorporated into MOFs either as building units of the MOF, such as a porphyrin linker [80,81], or by post-synthetic loading inside the pores [82]. It was shown that incorporating the photosensitive molecules in a separated organised lattice helps in increasing the quantum yield of ROS and prolong the excited triplet state of electrons, by minimising electron transfer through the π- conjugation in π–π stacking and hydrophobic forces that arise from their aggregation [83]. This antimicrobial mechanism is triggered by light and can provide us with another method of stimulated antimicrobial effect [84].

**Figure 10:**
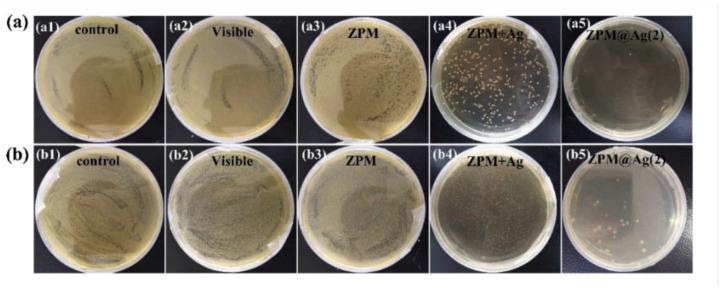
Agar plates showing the antimicrobial effect of Ag-doped ZPM MOFs against (**a**) *E. coli* and (**b**) *S. aureus.* Reproduced with permission from [88].

Ma et al. have used a hydrolytic-stable vanadium-based MOF, BIT-66, synthesised using VCl_3_ and 1,3,5-tris(4-carboxy-phenyl)benzene (H_3_BTB). The MOF showed bacteriostatic properties, because of its photocatalytic ability resulted from H_3_BTB which is a photosensitiser molecule [85]. The bacteriostatic effect was tested on *E. coli* colonies, where in the dark, after one hour, BIT-66 coating layers showed 44% removal efficiency, compared to 96%, in visible light. Another study from the same group showed that the widely used ZIF-8, exhibits antimicrobial photocatalytic action, with >99.9999% inactivation of *E. coli* under solar irradiation [86]. The incorporation of the photosensitiser molecule as a building unit of the MOF, as shown for ZIF-8, greatly enhanced the production of ROS, due to the metal cluster acting as a reservoir for photo excited electrons via metal-to-ligand charge transfer, thus prolonging the excited triplet state of electrons, in addition to being available for photocatalytic activity. The Zr-based MOF UiO-66-NH_2_ also showed photocatalytic activity, exhibiting peroxidase and oxidase mimetic activities, resulting in antimicrobial effect against *E. coli* [87]. There are multiple ways to improve the photoactivity of MOFs for antimicrobial applications, some of which involve structural modification, including the incorporation of noble metal nanoparticles. An example of this can be found in a study by Mao et al., where Ag nanoparticles were doped on zirconium-based porphyrinic MOF, ZPM [88]. After optimising with different amounts of Ag, an enhanced antimicrobial activity against *E. coli* and *S. aureus* were observed*,* as shown in Figure 10.

Metal substitution is another method to enhance the photoactivity of MOFs. Chen et al. have explored this by incorporating Ti^4+^ ions post-synthetically into the structure of pre-synthesised PCN-224 via cation exchange [89].

**Figure 11:**
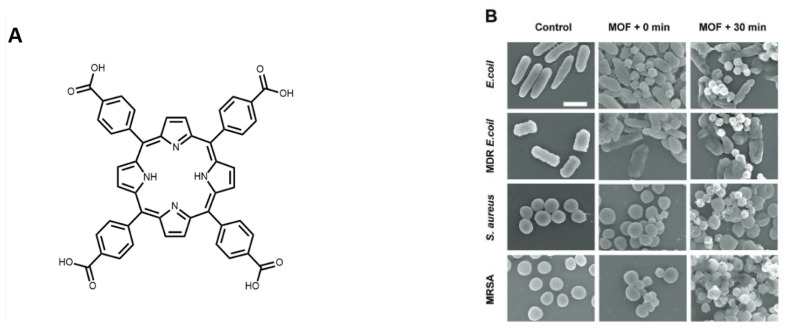
(**A**) The structure of the linker TCPP used for synthesis of PCN-224. (**B**) SEM images of bacterial morphology. Reproduced with permission from [90].

PCN-224 is a porphyrinic MOF, synthesised solvothermally using ZrCl_4_ and TCPP which is a photosensitiser (Figure 11). Although PCN-224 already exhibits photodynamic antimicrobial action, Chen et al. reported that the partial substitution of Zr(IV) by Ti(IV) enhanced the generation of ROS as the electron transfer from TCPP to Zr-Ti-oxo cluster improved over the Zr-oxo clusters [90,91]. Bactericidal efficacy was studied against Gram-negative and Gram-positive bacteria as well as their multi-drug resistant (MDR) isolates. After 30 min of irradiation, the MOF showed 96.4%, 96.8%, and 98.2% sterilization for MDR *E. coli*, Methicillin Resistant *S. aureus* (MRSA), and *S. epidermidis* (MRSE), respectively. The photoactivity of MOFs can also be enhanced by the integration of fluorescence energy transfer system, which often involves the use of hybrid-MOF materials. In 2022, Wang et al. synthesised hybrid ZIF-8 composites with Zn doped MoS_2_ [92].

Other post-synthetic enhancements involve chemical modification, such as sulphurisation. For example, in a study by Yu et al., CuS nanoparticles were embedded into the non-photoactive MOF, HKUST-1 via in situ sulphurisation, giving modified HKUST-1 the ability to absorb near infrared radiation (NIR) [93]. The evaluation of the photocatalytic activity was studied, showing that CuS embedded HKUST-1 showed catalytic activity under 808 nm NIR, as a result of localised surface plasmon resonance (LSPR) compared to no fluorescence for HKUST-1.

**Figure 12:**
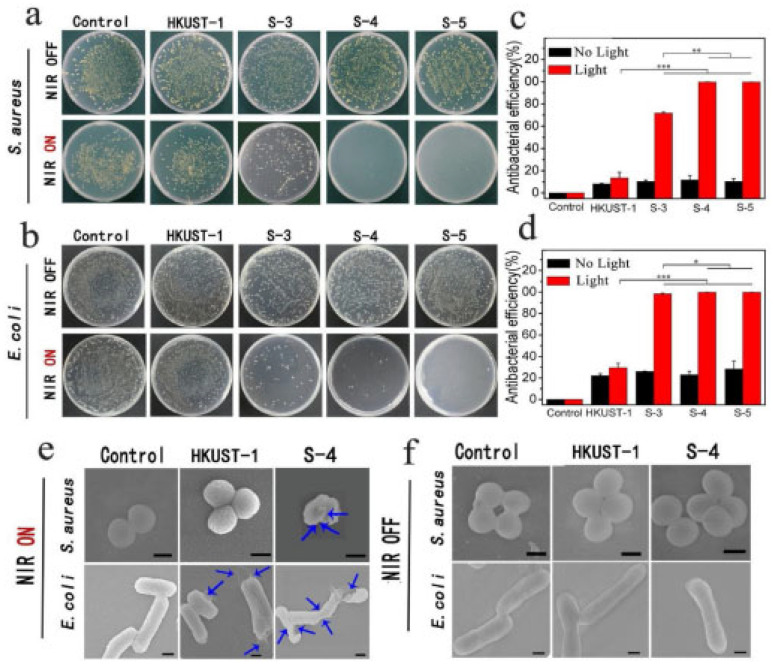
(**a**,**b**) In vitro antimicrobial studies. (**c**,**d**) %Antibacterial efficiency of MOFs against *E. coli* and *S. aureus*, respectively, error bars show ± standard deviation of * *p* < 0.05, ** *p* < 0.01, and *** *p* < 0.001. (**e**,**f**) Morphology of *E. coli* and *S. aureus* with NIR on and OFF, arrows in (**e**) pointing to the disrupted morphology of *E. coli* and *S. aureus* upon irradiation with NIR. Reproduced with permission from [93].

Antimicrobial activity of the CuS embedded HKUST-1 showed excellent disinfection ability, when irradiated under NIR for 20 min, of 99.70% and 99.80% for *S. aureus* and *E. coli*, respectively (Figure 12). The utilisation of photodynamic properties of MOFs, as well as their potential for enhanced antimicrobial effect, is drawing increasing attention. In addition, the antibacterial action of the MOFs can be enhanced by synergistic effect of loaded drugs and the building units of the MOFs, which are further enhanced by additional stimuli, such as photo-responsive units, allowing multiple parallel mechanisms to improve the antimicrobial effect.

### 3.3. MOFs as Chelating Agents

**Figure 13:**
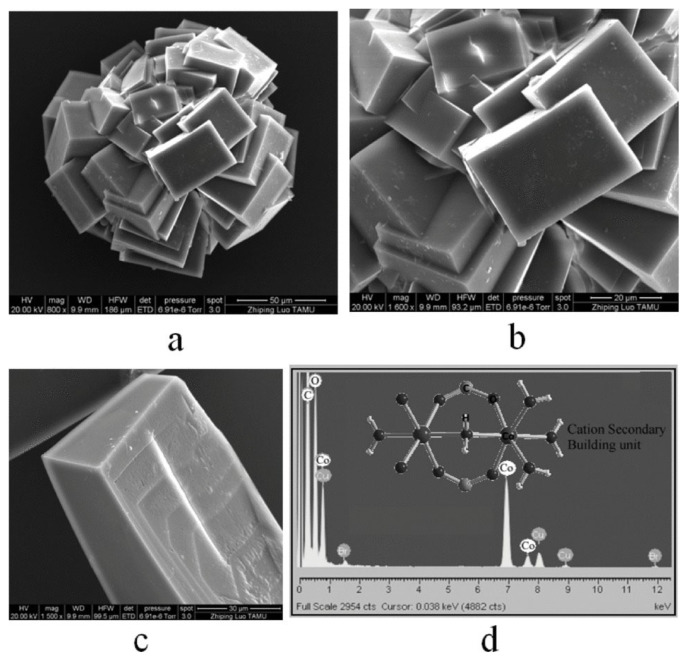
(**a**–**c**) SEM images of Co-TDM crystals; Scale bars: 50 μm, 20 μm, and 30 μm, respectively (**d**) EDS elemental analysis of Co-TDM with cation secondary building unit shown at the background. Reproduced with permission from [96].

Chelation reduces the positive charge on the metal ions, and therefore increases the lipophilicity, often by forming coordination bonds with aromatic ligands. The partial positive charge present on the metal component of the MOFs also helps to interact and facilitate their binding with the cell walls which are primarily lipophilic but contains many aminophosphates and carbonyl groups which act as potential coordinating sites [94]. Chelating mechanisms have been used in designing anticancer drugs, including the platinum-based drugs such as cisplatin that forms chelate with DNA, or as a detox agent, for the removal of excessive amounts of toxic metals present in the gastrointestinal contents. Several studies have proved the antimicrobial effects of chelate complexes. As shown in Table 1, there are several studies where chelation plays an important role for the antimicrobial action of the MOFs. In a study by Jo et al., a number of Cu-based MOFs have been reported to exhibit antimicrobial properties [95]. In addition, comparison of optical density and MBC showed that the MOFs had better antibacterial efficacy than their individual constituents, as the metal coordination sites can contribute towards antibacterial properties. The antimicrobial mechanism was primarily attributed to chelation of Cu^2+^ ions from the MOF. Coordinated MOF structure also shows higher lipophilicity than its separate constituents leading to better transport of the antimicrobial agents across lipid cell membranes. Another study by Zhuang et al. proposed chelation to be among several antimicrobial mechanisms of MOFs [96]. In their study, a Co-based MOF (Co-TDM) with an octa-topic linker was synthesised (Figure 13). Antimicrobial studies of this MOF exhibited an MBC of approximately 10–15 ppm against *E. coli* strains, in less than 60 min of incubation. The MOF-based composite showed 100% recyclability for bactericidal effect with excellent stability for more than 4 weeks. Among several proposed mechanisms of action, chelation is speculated to disable iron-related sulphur enzymes, inhibiting critical biosynthesis processes, by interacting with the ISC/SUF system and therefore causing toxicity [97].

Hence, in MOFs, the chelation mechanism plays an important role due to the abundance of active exposed metal centres, interacting with the structures of the microbe.

### 3.4. MOFs as Physical Disinfectants

**Figure 14:**
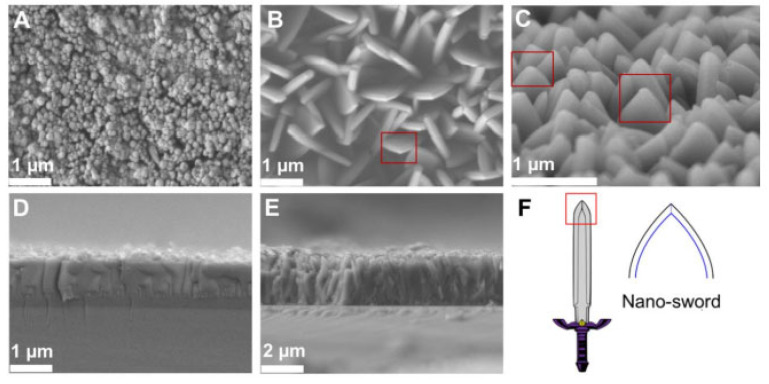
SEM images of ZIF-coated glass: (**A**,**D**) top view and side view of 2-MeIm:Zn of 35:1; (**B**,**C**,**E**) top, tilted, and side view of 2-MeIm:Zn of 7:1; red rectangle showing edge of the dagger-like structure (**F**) Illustration showing the dagger-like structure of ZIF-L with a ratio of 2-MeIm:Zn of 7:1. Reproduced with permission from [98].

Mechano-antibacterial effects rely on the surface morphology, size, and physical attributes of the nanoparticle. Given the variable nature of MOFs, slight variation in particle size, shape and crystallinity can be controlled via controlling the synthesis conditions. Physical disinfection strategies have been used to produce sterile surface, that can inhibit bacterial growth by preventing their adherence to the surface, Additionally, nanostructures can exhibit active contact-killing properties. Yuan et al. have reported leaf-shaped crystals of ZIF synthesised by altering ligand (2-methylimidazole or 2-MeIm) to metal (Zn^2+^) ratio [98]. SEM images of the positively charged ZIF nano-daggers are shown in Figure 14. The ZIF-coated surfaces exhibited antimicrobial effects against *E*. *coli*, *S. aureus*, and *C. albicans*. Several experiments were performed to rule out other antimicrobial mechanisms that might contribute to this effect, including leaching of Zn^+2^ and chemical interactions. The morphological change of the microbes in contact with the nano-dagger-coated surfaces were studied using SEM, showing significant deformation for all three microbes in 3 h, killing all cells within 20 h. This is a type of contact-based killing where the antimicrobial action is purely physical, and it depends on factors such as surface charge. In this study, the nano-dagger-shaped ZIFs exhibited a surface charge of +29 mV, and hence electrostatic interaction with negatively charged microbial cells can induce structural deformation. In addition, the hydrophobic nature and high water contact angle of 105.9° result in higher pressure being exerted upon contact, thus rupturing the cell membranes.

**Figure 15:**
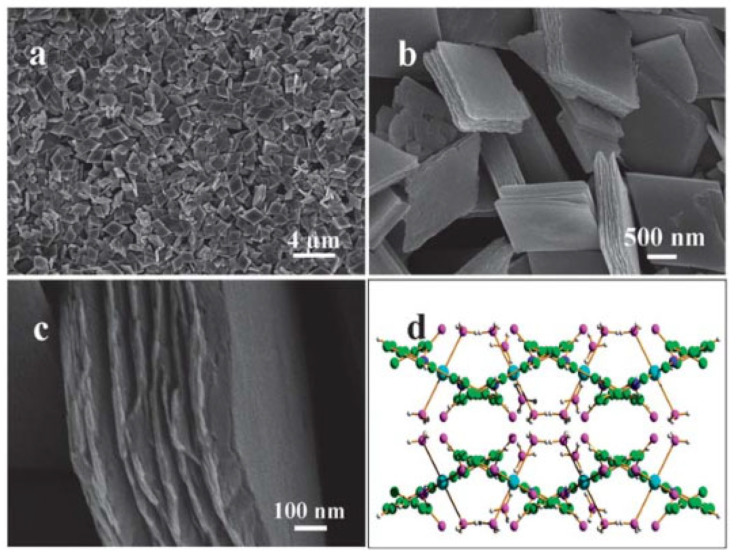
(**a**–**c**) SEM images of different morphologies and (**d**) crystal structure of Cu-based coordination polymer reported by Tynan et al. Reproduced with permission from [99].

In another study, the antimicrobial effects of different particle morphologies were explored for Cu-based coordination polymers [99]. The addition of Et_3_N or acetic acid during the synthesis of coordination manipulated the particle size and shape. In this study, several shapes of the polymers were synthesised, such as rhombus layers, disks, and bread-like structures (Figure 15). Antimicrobial studies have demonstrated different MIC values for each of the structures. For example, for *E. coli*, rhombus lump-shaped particles exhibited an MIC of 6.25 µg mL^−1^, while having an MIC of 12.5 µg mL^−1^ for rhombus layer shapes, and 50 µg mL^−1^ for both disks and bread-like structures, respectively. The full mechanism of antimicrobial effect is still unclear. However, it is obvious that the morphology of the particles played a significant role. Due to the customisable nature of MOFs, they possess great potential as platform for developing physical disinfectants, due to their unique crystal shapes, arrangement, and functional groups. Physical disinfection provides a drug-free alternative, which can subsidise the need for chemical agents that result in AMR. As shown in the following sections, MOFs can be further incorporated into polymer composites and fabrics, as will be shown below, to induce antimicrobial properties and play a role in developing physical disinfectants.

### 3.5. Other Mechanisms of MOFs Anticancer and Antimicrobial Action

In this section, other mechanisms of the antimicrobial action of MOFs are discussed. Sonodynamic therapy (SDT) has been recently studied as a non-invasive technique for the killing of cancer cells [100]. SDT is advantageous over other stimuli-responsive mechanisms such as PDT, as it has a higher tissue penetration ability to reach deeper without invasive damage to the surrounding tissues. SDT sonosensitisers are stimulated by ultrasound, and facilitate the production of ROS, hence inducing apoptosis. Many metal oxides, and sonosensitiser molecules such as emodin, curcumin and methylene blue are used for anticancer sonodynamic therapy. For efficient sonodynamic action, a good supply of oxygen must be present to facilitate the production of ROS. In a study by Geng et al., modified ZIF-8 was loaded with a sonosensitiser, hematoporphyrin monomethyl ether (HMME) [101]. The MOF was further decorated with F127 (non-ionic surfactant, to improve hydrophobicity), and treated with haemoglobin to yield HFH@ZIF-8. It was then further oscillated in a shaker to introduce oxygen to yield oxygen-carrying HFH@ZIF-8 which showed significant in vitro inhibition of MRSA upon irradiation with ultrasound (US), with a CFU of <5 × 10^3^ CFU g^−1^. The antimicrobial in vivo capabilities were demonstrated on MRSA myositis-bearing mice. The nanoparticle distribution was studied upon intravascular injection (IV) of a dose of 5 mg kg^−1^ in 200 µL solution at the tail vein. The HMME concentration was determined by in vivo imaging system (IVIS) to measure the biodistribution of the MOF composites. HFH@ZIF-8 showed higher accumulation in the MRSA-infected leg, indicating good targeting ability. MRSA-induced myositis was successfully treated with HFH@ZIF-8, showing highest antimicrobial effect and smallest oedema for US irradiated samples at 15 days.

The use of MOFs has been shown to increase the sonodynamic effect in some cases. For example, TiO_2_ is known to have poor sonodynamic effects due to the wide band gap. However, it was shown by Pan et al. that TiO_2_ incorporated in ZIF-8 (ZTN) increased the overall sonodynamic effect [102]. Antimicrobial studies (in vitro) demonstrated the sonodynamic antimicrobial action via confocal laser scanning microscopy (CLSM), showing all US irradiated MOF-treated cells to be dead, compared to other groups, such as MOF treated (without US), and the control. SEM imaging of the irradiated MOF-treated samples showed to have significant disturbed morphology. The study provided a unique approach to delivering the antimicrobial effect against MRSA that causes pneumonia, by utilising the ZIF-8 derived, TiO_2_ functionalised nanoparticles into inhaled dosage form. This suggests that MOFs might be advantageous to other inhaled dosage forms, by showing the site-specific antimicrobial effect via SDT. Biosafety studies on human (human umbilical vein endothelial cells (HUVEC)) and mouse cells (mouse fibroblast cells (NIH-3T3)) were carried out to determine biocompatibility, showing no signs of toxicity at concentrations below 100 µg mL^−1^. In vivo studies on immunocompetent mice with pneumonia showed significant antibacterial inhibition in organ homogenates at 2, 4 and 6 days of 84.8%, 77% and 80.0%, respectively. No bacteria were observed for MOF-treated mice in liver or spleen, indicating complete eradication of *K. pneumonia.* On immunocompromised mice, bacterial dissemination was observed in day 2, with US-treated ZTN having the highest antimicrobial action. No pathological signs of organ damage were observed upon the pulmonary administration of the MOFs.

Another example of the enhancement of sonodynamic effect can be found in a study by Yu et al. where atom-doped, pyrophyrin-based MOF, HNTM-Pt@Au was used [103]. In this study, the introduction of single metal ions improved the absorption of oxygen, and hence enhanced the production of ROS. The US-irradiated porphyrin component of the MOF transcended into an excited state, and then reached a triplet state through intersystem crossing. The excited electrons that resulted from this process were transferred to the metal ion, which then converted oxygen to a reactive singlet state. In addition to the excitation by US, the porphyrin-based MOF is also a photosensitiser, allowing for a multi-stimulus trigger of ROS. In this in vitro study, the colony counting method showed that the use of HNTM-Pt@Au under US irradiation showed a 99.93% inhibition of MRSA, compared to 97.86% for non-doped HNTM-Pt, which showed obvious enhancement of antimicrobial activity as a result of doping. In vitro biocompatibility studies showed good cell viability; however, this decreased under US irradiation, due to the generation of ROS. The MOFs were tested for their ability to eradicate osteomyelitis for MRSA-infected rat tibia. The functionalised MOFs were successful in treating the infection, with significantly decreased inflammatory response.

MOFs can also possess enzyme-mimetic antimicrobial action through the partially positive charge on the metal ion centres, by providing a site for catalytic reactions. In some studies, it was already shown that due to this, some MOFs possess peroxidase-like activity and hence help generate the production of ROS [104,105]. The presence of surface-active metal sites on MOF structure was utilised for its antibacterial action by Lee et al. [106]. In this study, a Cu-BTC MOF-based composite showed in vitro antimicrobial effect against *P. aeruginosa*, *K. pneumonia* and MRSA of reduction percentage of 97.8%, 99.3% and 77.6%, respectively. It was proposed that the role of the active Cu^2+^ centres in the MOF was more significant in its antimicrobial efficiency, as factors such as disintegration of the MOF was ruled out by testing the leaching of Cu^2+^ and proving the stability of the Cu-BTC framework. The metal sites are able to catalyse the production of ROS through Fenton-like reactions [104], and such an approach is called chemodynamic therapy.

Another interesting study incorporated the silk sericin as a linker in a Cu-based MOF, Cu-SER [107]. In vitro antimicrobial activity against *E. coli* and *S. aureus* was studied for this MOF by measuring the zeta potential of the bacterial surfaces. It was reported that the MOF-treated samples showed a change in the zeta-potential on the bacterial cell membrane, hence disrupting it. This change was further observed by SEM as the morphology of the cells significantly changed. This mechanism was attributed to surface neutralisation. Neutralisation-mediated killing occurs when cationic antimicrobial peptides (AMP) initiate electrostatic interactions with the negatively charged lipopolysaccharides (LPS) on the bacterial surface, resulting in a change in the bacterial surface charge and disabling of the cell membrane [108]. Silk sericin is a biocompatible protein with antimicrobial properties [109], and in this study its inherent antibacterial action was utilised into MOFs. The study further suggests that the slow release of Cu^+2^ might also have contributed to the antimicrobial effect. Cell viability studies on human adipose-derived stem cells (hASCs) showed highest viability at concentration of 62.5 μg mL^−1^. When treated with osteogenic media for 7 days, the cells showed characteristic fibroblast phenotype with actin stress fibres present in the cytoplasm. The actin fibres showed a crisscross pattern, resembling those of mesenchymal stem-cell-derived osteoblasts. This actin arrangement correlates with osteogenesis, hence demonstrating the pro-osteogenetic ability of Cu-SER. this study demonstrates the potential of MOFs in clinical setting, the biocompatible Cu-MOFs can act as template during the biomineralisation and proliferation of human bone tissue, hence offering a solution to osteomyelitis resulting from tooth decay or broken bone.

**Figure 16:**
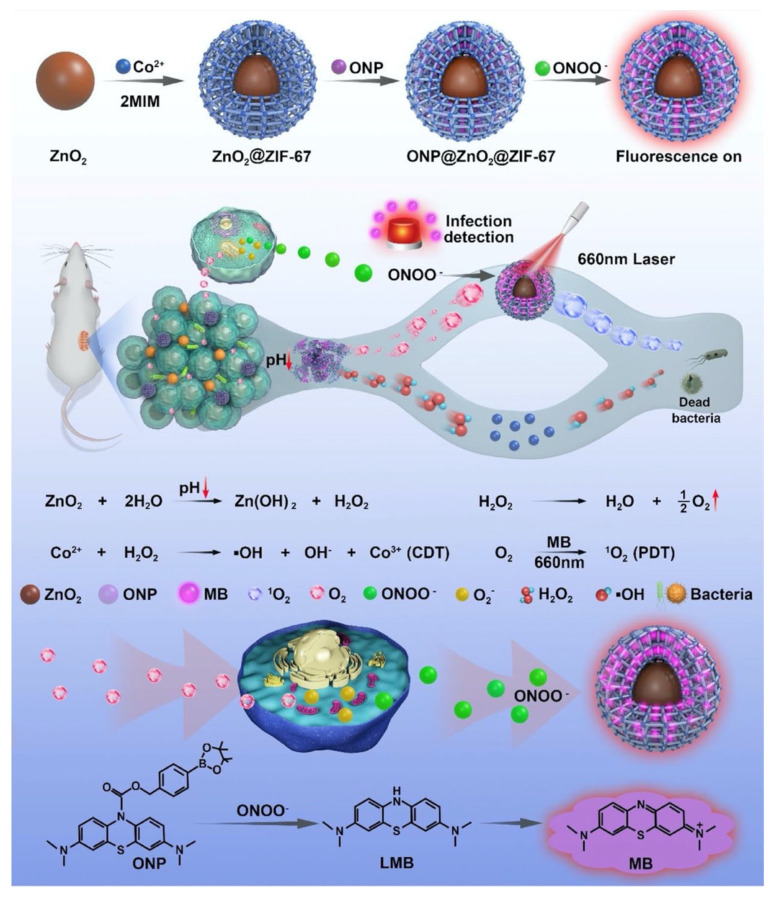
Scheme showing preparation of ONP@ZnO_2_@ZIF-67 and the PDT- and CDT-induced mechanism of antibacterial action for the material, showing the ZnO core coated with ONP-loaded ZIF-8 MOFs. Upon being exposed to ONOO^−^_,_ MB would be generated from ONP, hence exhibiting fluorescence. MB generation can be used as a marker for infection sites for detection, as a result of the over expression of ONOO^−^ and low pH at infection sites. When irradiated, the nanoparticles exhibit antibacterial properties as a result of ROS generation. Reproduced with permission from [110].

As it becomes obvious from the above examples, the antimicrobial effect of MOFs cannot be explained by a single mechanism. There is a wide range of antimicrobial mechanisms that MOFs can take advantage of. For example, their customisable structures, post-synthetic modifications, and integration with other materials into composites broaden their scopes for antimicrobial action. In a study in 2022, Huang et al. used a ZIF-67 armoured zinc peroxide core–shell structure with a loaded organic NIR probe (ONP) as an Infection detecting and remediating platform (Figure 16) [110]. ZIF-67 was functionalised with a zinc-peroxide structure, loaded with a methylene blue (MB)derivative as an ONP. In sites of infection, a higher concentration of peroxynitrile (ONOO^−^) is usually present. The ONP fluorescent probe would react with ONOO^−^, yielding MB. MB acts as a photosensitiser, with the ability to generate ROS upon irradiation in ONOO^−^ rich infection sites to kill the bacterial cells. Additionally, through the Co^2+^ active sites in the structure of ZIF-67, the nanoparticles also catalyse decomposition of H_2_O_2_ into reactive OH radical species through Fenton-like reactions. In vitro antimicrobial activity was studied on MRSA as well as antibiotic resistant *E. coli* and viability of MRSA was found to decrease in acidic environments, like that found at infection sites. The majority of MRSA was eradicated at concentration of 25 μg mL^−1^ under irradiation of 660 nm light. Cytotoxic and haemolytic assays on Murine L929 and RAW 264.7 cells using the CCK-8 Kit and blood of healthy BALM/c mice demonstrate the biocompatibility of the nanoparticles in vitro. Cell viability experiments showed the nanoagents as biosafe up to concentration of 25 µg mL^−1^. In addition, at concentration below 100 µg mL^−1^, no haemolysis of red blood cells was observed. Pathological examination of the heart, spleen, kidney, and liver showed no signs of inflammation or abnormality in vivo. The nanoparticles demonstrated their in vivo antibacterial effect on infected skin wounds. Large abscesses were observed in the control group that were treated with PBS at day 15, while the nanoparticle-treated groups showed better recovery, with increasing the average skin recovery to 98%. This study shows the synergistic antimicrobial effect of chemodynamic therapy (CDT) and PDT, producing OH and ^1^O_2_ species, respectively.

**Table 1:** Summary of MOFs studied for antimicrobial applications.

MOF	Antibacterial Agent	Mechanism of Action	Target Organism	Loading Capacity (%)	Clinical Significance	Antimicrobial Efficacy	Ref./Year
Mn-MOF Mg-MOF	nalidixic acid	MOF disintegration	*E. coli* *S. aureus* *E. faecalis* *C. albicans* *S. cerevisiae*	-	Cytotoxic assay on human colorectal adenocarcinoma CaCo-2 cell. No significant effect on cell viability	In vitro Higher antimicrobial activity than for nalidixic acid	[74] (2019)
ZIF-8	chloramphenicol	Drug release	*E. coli* *S. aureus*	32.58 ± 2.65	-	In vitro >99.9% growth reduction in 24 h.	[111] (2022)
MIL-101(Fe)	Ag^+^	Drug release	*E. coli* *S. aureus* *S. epidermidis* *A. cereus* *A. jungii* *P. aeruginosa*	0.0127	No severe haemolytic behaviour. AD293 cell-viability studies reveal MOF non-toxic and hypotoxic	In vitro Inhib. zones (mm): 12 12.3 10 11 11 11	[67] (2022)
MIL-53(Fe)	vancomycin	Drug release	*S. aureus*	20	Biocompatible, promote osteogenic differentiation and proliferation of MC3T3 cells Cytotoxic studies using MTT assay prove non-toxic	In vitro Antibacterial ratio up to 90%	[112] (2017)
UiO-66	ciprofloxacin	Drug release	*E. coli* *S. aureus*	84	-	In vitro Inhib. zones: 24 mm 22 mm	[60] (2019)
ZIF-8	ceftazidime	Drug release MOF disintegration	*E. coli*	10.9	Cell viability studies on human lung epithelial cell line (A549) and mouse macrophage cells lines RAW 264.7 cells show dose-dependent toxicity	In vitro Less bacterial growth when exposed to loaded MOFs	[65] (2019)
ZIF-8	gentamicin	Drug release	*E. coli* *S. aureus*	19	Cytotoxicity studies on human Caucasian foetal foreskin fibroblast (HFFF2), increase of 75% in cell viability when incubated with 10–30 μg mL^−1^ concentrations of MOF for 48 h	In vitro Inhib. zone: <14 mm 12 mm	[72] (2018)
ZIF-8	ciprofloxacin	Drug release	*E. coli* *S. aureus*	21	-	In vitro Inhib. zone: 46 mm 49 mm	[113] (2017)
Zn_2_(bdc)_2_(dabco)	gentamicin	Drug release	*E. coli* *S. aureus*	14 (from TGA)	-	In vitro Inhib. zone: 9 mm 16 mm	[70] (2018)
ZIF-8	rifampicin 2-nitrobenzealdehyde Zn^2+^	Light-triggered drug release MOF disintegration	*E. coli* *MRSA*	-	MTT assay on Hela cells prove MOF had no signs of cytotoxicity. Promotion of scar generation in mice injury model	In vitro: concentration and illumination-time-dependent effect. Optimum conditions: 10 μg mL^−1^ and 120 min illumination time In vivo: Mice injury model Wound infection size in mice decreased by 80% through synergistic treatment	[114] (2018)
ZIF-8	vancomycin folic acid	Drug release	*MDR S. aureus* *E. coli*	24	-	In vitro MIC: 8 µg mL^−1^ 16 µg mL^−1^	[115] (2017)
Ag-MOFs	organic radical anions Ag^+^	MOF disintegration photochromism	*E. coli* *P. aeruginosa* *B. subtilis* *S. aureus* *MRSA* *MDR-PA*	-	-	In vitro: inhibition of more than 98.47% of drug-resistant bugs in vivo: better healing of MDR-PA infected wounds in mice injury model	[116] (2022)
IRMOF-3 MOF-5 Zn-BTC	ampicillin kanamycin	Drug release	*S. aureus* *S. lentus* *L. monocytogenes* *E. coli*	-	Cytotoxic assay on human dermal non-cancerous (HaCaT) cells using MTT assay. Low toxicity for Zn-BTC MOFs and MOF-5/ampicillin Moderate toxicity for IRMOF-3 and MOF-5/kanamycin	In vitro Enhanced antimicrobial effect against Gram-negative and Gram-negative bacteria	[76] (2018)
PCN-224(Zr/Ti)	PCN-224 Ti	Photodynamic therapy	*MDR E. coli* *MRSA* *C. baumannii* *MDR A. baumannii* *MRSE*	-	Biocompatibility studies on human umbilical vein endothelial cells (HUVECs) show 90% of cells maintained vitality In vivo biological safety on mice studies through IV injection indicate negligible biotoxicity	In vitro 96.4%,96.8% and 96.2% sterilization for MDR *E. coli*, MRSA and MRSE, respectively	[90] (2022)
PCN-224(Cu/Ti)	PCN-224 Cu	Photodynamic therapy (photocatalysis)	*S. aureus*	-	Cytotoxicity studies on mouse NIH-3T3 cells indicate MOFs are not cytotoxic In vivo toxicity evaluation show no organ damage	In vitro Highest efficacy 99.71% within 20 min of irradiation. In vivo wound-infection healing show high antimicrobial action and accelerated wound healing	[117] (2020)
ZIF-8	physicon	Drug release	*P. putida* *E. coli* *Eng. E. coli* *S. aureus*	11.49%	-	In vitro Inhib. zones: 13 mm 23 mm 18 mm 20 mm	[118] (2019)
(MIL-101-based MOFs) Fe-101 Al-101 Fe-88	indocyanine green	Drug loaded, Photodynamic therapy	*E. faecalis*	16.93 ± 0.32% 18.17 ± 0.31% -	In vivo studies in infected tooth show decreased gene expression of *E. faecalis*	In vitro Red. in biofilm formation 47.01% 53.68% 37.54%	[119] (2018)
ZIF-8	Zn^2+^	MOF disintegration	*E. coli*	-	-	In vitro Cell reduction of 4.79 in log_10_	[71] (2021)
PEI-Ce (III) MOFs	Ce^3+^ PEI	Peroxidase-like activity	*E. coli* *P. aeruginosa* *B. subtilis* *S. aureus* *C. albicans*	-	Human blood compatibility tests: PEI-Ce(III) MOF: non-haemolytic, non-coagulative p-PEI-Ce(NO_3_)_3_ MOFs: slightly haemolytic and non-coagulative	In vitro MBC ranging from 1.25–5 mg mL^−1^	[120] (2022)
Ce-MOF	Ce-MOF	Enzyme-mimetic activity	*A.flavus* *A.niger* *A. terreus* *C. albicans* *R.glutinis*	-	-	In vitro 93.3–99.9% inhibition efficiency	[121] (2020)
CuTCPP-Fe_2_O_3_	ROS	Photodynamic therapy	*P. gingivalis* *F. nucleatum* *S. aureus*	-	No signs of organ damage in vivo In vivo- Higher antibacterial effect than minocycline and vancomycin for MOF-treated samples in periodontitis mice model Reduced inflammation, promoted angiogenesis	In vitro 99.87 ± 0.09% 99.57 ± 0.21% 99.03 ± 0.24%	[122] (2022)
BIT-66	ROS	Photocatalysis	*E. coli*	-	-	In vitro 44% and 96% removal efficiency in dark and light, respectively	[85] (2020)
ZIF-67 Co-SIM-1 AgTAZ	Co^2+^ Ag^+^	MOF disintegration	*E. coli* *P. pudita* *S. cerevisiae*	-	-	In vitro Inhib. zone of mostly around 15 mm, except for AgTAZ, 2 mm. -3 month antibacterial effect	[123] (2014)
MIL-53(Al) NH_2_-MIL-53(Al)	Al^+^	MOF disintegration	*E. hirae*	-	-	In vitro MIC: 8 mg L^−1^	[124] (2022)
VAC-Zn-BTC-coordination polymer	Zn-BTC vancomycin	MOF disintegration Drug release	*MRSA*	-	Cytotoxicity assay on human alveolar basal epithelial cells (A549), embryonic kidney cells (HEK-293) and human breast cancer cell line (MCF-7) show MOF non-toxic at conc. <80 μg mL^−1^. Low haemolytic activity.	In vitro MIC: 1.02 μg mL^−1^	[125] (2022)
Cu-MOF	ROS	Photodynamic therapy	*E. coli* *S. aureus*	-	Cytotoxic assay on adenocarcinomic human alveolar basal epithelial cancer cells (A549) show significant photocytotoxicity when irradiated (IC_50_: 15.9 mg mL^−1^) with minimal dark cytotoxicity (IC_50_: 225 mg mL^−1^) Interaction with blood serum albumin (BSA)	In vitro MIC: 300 μg mL^−1^ MIC: 350 μg mL^−1^	[84] (2022)
HFH@ZIF-8	HMME	Cargo release Sonodynamic therapy	*MRSA*	-	In vivo biodistribution in myositis-bearing mice show inflammation targeting property. effective treatment of myositis in mice	In vitro Average bacteria colony number < 5 × 10^3^ CFU g^−1^ In irradiation of US and O_2_	[101] (2022)
HKUST-1	Cu^2+^	MOF disintegration	*S. cerevisiae* *G. candidum*	-	-	In vitro Complete inhibition of *S. cerevisiae* Decrease from 6.16 to 1.29 CFU mL^−1^ for *G. candidum*	[126] (2012)
TA-MOF	-	-	*A. fumigatus* *F. oxysporum* *R. equi* *S. epidermidis* *S. dysenteriae* *E. coli*	-	-	In vitro MIC: 64 μg mL^−1^ MIC: 32 μg mL^−1^ MIC: 16 μg mL^−1^ MIC: 32 μg mL^−1^ MIC: 128 μg mL^−1^ MIC: 32 μg mL^−1^	[127] (2022)
[Zn(dicarb)_2_]·2H_2_O	Zn^2+^	MOF disintegration	*S. aureus* *S. equinis* *B. cereus* *A. baumannii* *K. pneumoniae* *C. albicans* *F. oxysporum*	-	-	In vitro MIC: 64 μg mL^−1^ MIC: 32 μg mL^−1^ MIC: 512 μg mL^−1^ MIC: 256 μg mL^−1^ MIC: 64 μg mL^−1^ MIC: 128 μg mL^−1^ MIC: 512 μg mL^−1^	[128] (2021)
([Zn(μ-4-hzba)_2_]_2_·4(H2O))_n_	μ-4-hzba Zn^2+^	MOF disintegration	*S. aureus*	-	-	In vitro Inhib. Zone: 14.6 ± 3.1 mm	[129] (2017)
Cu-BTTri	Cu^2+^	MOF disintegration	*P. aeruginosa*	-	-	In vitro 85% reduction in bacterial attachment	[130] (2017)
ZIF-8	ROS	Photocatalysis	*E. coli*	-	-	>99.9999% inactivation efficiency	[86] (2019)
PCN-134-2D	ROS artemisinin	Photocatalysis Artemisinin production	*-*	-	-	-	[42] (2019)
UiO-66-NH_2_	ROS	Photocatalysis Peroxidase-mimetic action	*E. coli*	-	In vitro cytotoxicity studies on mouse NIH-3T3 cells using MTT assay prove non-toxic	-antimicrobial activity in presence of UV light	[87] (2021)
HKUST-1	CuS	Photothermal	*S. aureus* *E. coli*	-	In vitro cytotoxicity studies on mouse NIH-3T3 cells using MTT assay show cells viability ranging from 60% to 70% without NIR.	In vitro 99.70%, 99.80% inhibition	[93] (2022)
ZPM@Ag	Ag^+^ ROS	Photoactivation Ag^+^ release	*S. aureus* *E. coli*	-	-	In vitro 2.4% and 0.3% viability	[88] (2021)
Zn–MoS_2_-ZIF-8	ROS Zn^2+^	Photocatalysis MOF disintegration	*S. aureus*	-	In vitro cytocompatability on NIH-3T3 cells show 49.56% survival rate upon irradiation Successful treatment of wound in mice after 10 days No damage of major organs found	In vitro 99.7% antibacterial efficacy under 660 nm irradiation	[92] (2022)
GS5-CL-Ag@CD-MOF	Ag^+^ release	MOF disintegration Drug release	*E. coli* *S. aureus*	-	In vitro haemostatic studies In vivo wound healing experiment show down regulation of cytokines and inflammatory response, promote healing	In vitro MIC: 16 µg mL^−1^ For *E. coli*	[131] (2019)
ZAG NPs (ZIF-8-derived nanoenzyme)	Zn^2+^ release Au NP GOx	Drug release MOF disintegration	*E. coli* *S. aureus*	-	In vivo anti-inflammatory action, enhanced wound regeneration	In vitro MIC: 8 µg mL^−1^ 4 µg mL^−1^ In vivo: rapid sterilisation of S. *aureus*-infected wounds	[132] (2022)
transition metal complexes	-	Chelation effect	*S. aureus* *C. albicans* *B. subtilis* *E. coli* *P. aeruginosa*	-	-	In vitro MIC between 6.25 and 50 µg mL^−1^	[133] (2012)
([Ni(μ1,5-dca)_2_(μ-hmt)]H_2_O)_n_	-	Chelation effect	*K. pneumonia*	-	-	In vitro MIC: 16.9 µM	[134] (2015)
[Cu(C_5_H_4_O_4_)_2_(C_6_H_6_N_2_O)_2_(H_2_O)_2_·2(H_2_O)]	-	Chelation effect	*E. coli* *S. aureus* *P. aeruginsoa*	-	-	In vitro MIC: 0.0004 g L^−1^ 0.00006 g L^−1^ 0.0016 g L^−1^	[135] (2016)
Cu-MOFs	-	Chelation effect	*E. coli* *S. aureus* *K. pneumonia* *P. aeruginosa* *MRSA*	-	-	In vitro MBC of 20 μg mL^−1^	[95] (2019)
[Cu(L)_2_Cl_2_]	-	Chelation effect	*E. coli* *K. pneumonia* *S. aureus* *B. subtilis*	-	-	In vitro Inhib. zone: 19 mm 28 mm 24 mm 20 mm	[136] (2010)
[Cu(L-Arg)_2_(µ-4,4‘-bpy)]Cl_2_·3H_2_O]_∞_	-	Chelation effect	*S. mutans* *E. hirae* *B. subtilis* *S. aureus* *P. aureginosa* *E. coli* *S. enterica* *S. flexneri* *S. cerevisiae* *C. albicans*	-	-	In vitro MIC is <15 M	[137] (2015)
Co-TDM	-	Chelation effect	*E. coli*	-	-	In vitro MBC ranging from 10 to 15 ppm	[96] (2012)
Cu-BTC	surface active Cu^2+^	Fenton-like reaction	*P. aeruginosa* *K. pneumoniae MRSA*	-	In vitro cytotoxicity studies on mouse embryonic fibroblast (MEF) cells. MEF viability over 95%	97.8%, 99.9% and 77.6% reduction, respectively.	[106] (2021)
Bi-MOFs	-	Chelation effect	*E. coli* *E. aerogenes* *S. aureus* *B. cereus* *C. butyricum*	-	-	In vitro Highest microbial effect was against *B. cereus* an *C. butyricum*	[138] (2019)
[(AgL)NO_3_] 2H_2_O [(AgL)CF_3_SO_3_] 2H_2_O [(AgL)ClO_4_] 2H_2_O	Ag^+^	MOF disintegration	*E. coli* *S. aureus*	-	-	In vitro Inhib. zones ranging from 13 to 19 mm	[139] (2010)
[Ag(Bim)] [Ag_2_(NIPH)(HBim)] [Ag_6_(4-NPTA)(Bim)_4_] [Ag_2_(3-NPTA)(bipy)0.5(H_2_O)]	Ag^+^	MOF disintegration	*E. coli* *S. aureus*	-	-	In vitro MIC values ranging from 5 to 20 ppm	[140] (2014)
[Ag(L1)](NO_3_) [Ag(L2)]_n_(NO_3_)_n_(H_2_O)	Ag^+^	MOF disintegration	*E. coli* *S. typhimurium* *K. pneumoniae* *S. marcescens* *S. aureus* *streptococcus*	-	-	In vitro Inhibition zones ranging from 14 to 26 mm	[141] (2018)
[Ag_2_(O-IPA)(H_2_O)·(H_3_O)] [Ag_5_(PYDC)_2_(OH)]	Ag^+^	MOF disintegration	*E. coli* *S. aureus*	-	-	In vitro MIC of 5–10 and 10–15 ppm for *E. coli*, and 10–15 and 15–20 ppm *for S. aureus*	[142] (2014)
[Ag(NO_3_)(μ3-PTA═ O)]n Ag_2_(μ2-SO_4_)(μ5-PTA═ O)(H_2_O)]_n_	Ag^+^	MOF disintegration	*E. coli* *P. aeruginosa* *S. aureus* *C. albicans*	-	-	In vitro MIC of *E. coli* and *P. aeruginosa* ranging between 6 and 7 μg mL^−1^.for *S. aureus* and *C. albicans* range 20–30 μg mL^−1^.	[143] (2011)
Ag-isonicotinic acid polyethyleneglycol complexes	Ag^+^	MOF disintegration	*S. epidermidis* *S. aureus* *E. coli* *P. aeruginosa*	-	-	In vitro Inhibition zones reached up to 12 and 15 mm	[144] (2010)
[Ag(µ3-PTA=S)]_n_(NO3)_n_·nH_2_O Ag_4_(µ4-PTA=S)(m5-PTALS)(µ2-SO_4_)_2_(H_2_O)_2_]_n_ 2nH_2_O (BioMOFs)	Ag^+^	MOF disintegration	*E. coli* *S. aureus* *P. aeruginosa* *C. albicans*	-	-	In vitro Most active agaist Gram-negative bacteria (MIC of 4–5 μg mL^−1^) MIC for *S. aureus* 20 μg mL^−1^	[145] (2013)
Ag(I) 1,3,5-Triaza-7-phosphaadamantane coordination networks	Ag^+^	MOF disintegration	*E. coli* *S. aureus* *P. aeruginosa* *C. albicans*	-	-	In vitro MIC for *E. coli* and *C. albicans* ranging from 6−7 and 30−50 μg mL^−1^	[146] (2014)
[[Ag_4_(μ4-pzdc)_2_(μ-en)_2_]·H2O]_n_	Ag^+^	MOF disintegration	*E. coli* *S. aureus* *C. albicans*	-	-	In vitro MIC range 18–63 µg mL^−1^	[147] (2012)
Ag_3_(3-phosphonobenzoate)	Ag^+^	MOF disintegration	*S. aureus* *MRSA* *E. coli* *P. aeruginosa*	-	In vitro haemolysis assay on human red blood cells shows MOfs did not exhibit haemolytic activity at conc. <500 µM	In vitro MBC value from 20 to 75 µM	[148] (2011)
Cu-SURMOF-2	Cu^2+^	MOF disintegration	*C. marina*	-	-	In vitro 88% damaged organisms	[149] (2013)
BioMIL-5 (BioMOF)	Zn^2+^ AzA	MOF disintegration	*S. aureus* *S. epidermidis*	-	-	In vitro MIC: 1.7 mg mL^−1^ 1.7 mg mL^−1^	[150] (2015)
Ni-MOF	Ni^2+^ Hmim	MOF disintegration	*(ESBL-1)* *(P. aeruiginosa)* *(MRSA ATCC 33591)* *(MRSA clinical strain N7)*	-	In vivo cytotoxicity assay on *A. Salina* by treating the feed (nauplii) with the MOFs. Negligible toxicity, at maximum dose of 150 μg mL^−1^	In vitro MIC:800 µg mL^-^1 ppm^−1^ MIC:1000 µg mL^−1^ ppm^−1^ IC_50_:15.19 ± 1.41 µg mL^−1^ IC_50_: 25.14 ± 0.75 µg mL-1	[151] (2020)
Fe(III)-MOF	Fe^3+^	MOF disintegration	*S. aureus* *E. coli* *Candida* spp. *A. niger*	-	-	In vitro Inhib. zones: 51 mm 38 mm 48 mm 52 mm	[152] (2022)
Cu-MOFs nanoparticles	-	Chelation Particle morphology	*S. aureus* *Candida* spp. *E. coli* *Pseudomonas* Sp. *Klebsiella* Sp.	-	-	In vitro Inhib. zone: (conc. 100 μg ml^−1^) 42 mm 46 mm 45 mm 49 mm 35 mm	[153] (2018)
ZIF-L	-	Particle morphology	*E. coli* *S. aureus* *C. albicans*	-		0% viability after 20 h of incubation	[98] (2017)
CD-MOFs	caffeic acid	Drug release	*E. coli* *S. aureus*	19.63 ± 2.53%	-	In vitro MIC: 25 mg mL^−1^ 25 mg mL^−1^	[154] (2022)
([Cu(dcbp)(H_2_O)2] 2H_2_O)_n_ (rhombus lump shape)	-	Particle morphology	*B. subtilis* *S. aureus* *S. enteriditis* *E. coli* *P. vulgaris* *P. aeruginosa*	-	-	In vitro MIC (µg mL^−1^): 12.5 12.5 25 6.25 12.5	[99] (2011)
ZIF-8-derived carbon@TiO_2_	TiO_2_	Sonodynamic therapy	*E. Faecium* *S. aureus* *K. pneumoniae* *A. baumannii* *P. aeruginosa* *Enterobacter*	-	Biosafety studies on human umbilical vein endothelial cells (HUVEC) and mouse fibroblast cells (NIH-3T3) show no signs of toxicity at concentrations 100 µg mL^−1^. In vivo studies on immunocompetent mice with pneumonia showed significant antibacterial inhibition. No pathological signs of organ damage. Inhalable dosage form	In vitro Inhibition of Gram-negative bacteria. 99.2%, 87.1%, 95.6%, and 81.5% inhibition of *K. pneumoniae, A. baumannii, P. aeruginosa*, and *K. aerogenes*, respectively	[102] (2022)
HNTM-Pt@Au	Au	Sonodynamic therapy	*MRSA*	-	In vitro biocompatibility studies showed good cell viability MOFs were successful in treatment of osteomyelitis with decreased inflammatory response.	In vitro 99.9% efficiency under 15 min of US irradiation	[103] (2021)
C-Ti-MOF (NH_2_-MIL-125(Ti) composite)	TiO_2_	Photodynamic therapy Photothermal therapy	*S. aureus*	-	Biosafety studies on human umbilical vein endothelial cells (HUVECs) show good biocompatibility	In vitro MIC: 0.16 mg mL^−1^	[155] (2022)
Ag NP-loaded Cu-BTC	Ag^+^	Drug release	*E. coli* *S. aureus*	1.76–2%	-	In vitro MIC ranging from 156.2 to 625 µg mL^−1^	[156] (2022)
MOF-74(Zn) MOF-74(Cu)	linezolid	Drug release	*S. aureus*	4.91% 1.75%	-	In vitro MIC: 75.0 µg mL^−1^ 32.0 µg mL^−1^	[157] (2022)
Ag-associated UiO66-Nap (4-sulfo-1,8-naphthalimide immobilised UiO66-NH_2_)	Ag^+^	-	*C. albicans* *E. coli* *P. aeruginosa* *K. pneumoniae* *MRSA* *S. enteriditis* *S. lutea* *B. cereus*		-	In vitro MIC of 0.019 mg mL^−1^ against Gram-positive and Gram-negative bacteria. High antifungal activity	[158] (2022)
UoB-6 (BioMOF-Mn)	cationic functional groups Mn^2+^	MOF disintegration Oxidative effect	*E. coli* *C. albicans*	-	-	In vitro MIC of 4096 and 2048 µg mL^−1^	[159] (2022)
Cu-MOF MCu-MOF	Cu^2+^	MOF disintegration	*B. subtilis* *L. cereus* *S. aureus* *E. coli* *S. enterica* *A. niger*	-	-	In vitro Inhibition zone ranging from 10 to 17 mm	[160] (2022)
Zn-MOF Cu-MOF Cu/Zn hybrid MOF	Cu^2+^ Zn^2+^	MOF disintegration	*E. coli* *S. enterica subsp. enterica* *P. mirabilis* *R. equi* *C. albicans*	-	-	In vitro MIC ranging from 32 to 1024 μg mL^−1^	[161] (2022)
Fe_3_O_4_/Zn-MOF	Zn^2+^	MOF disintegration	*P. aeruginosa* *S. dysenteriae* *S. agalactiae* *C. albicans*	-	-	In vitro MIC values for Gram-positive and Gram-negative bacterial strains, between 16 and 128 μg mL^−1^, and for fungal strain, 128 μg mL^−1^	[162] (2022)
MIL-100(Fe)	carvacarol	Drug loading, Redox activity	*E. coli* *L. innocua*	42	In vitro Cell viability studies on human embryonic kidney cells (HEK293). 100% cell viability when exposed to 200 μg/mL equivalent carvacrol for 24 h	In vitro Antimicrobial activity of films(log(CFU/mL)): 7.28 ± 0.1 7.64 ± 0.87	[163] (2022)
MOF-derived CuO composite Deposited with CuO/AgX (X = Cl, Br, or I)	ROS Cu^2+^ Ag^+^	Photocatalysis MOF disintegration	*E. coli* *S. aureus*	-	-	In vitro CuO/AgBr-15 has highest catalytic disinfection, followed by CuO/AgBr-15 and CuO/AgCl-15	[164] (2022)
MOF-NC (MOF nanocages)	Ag^+^ Zn^2+^ ascorbic acid	MOF disintegration Drug release	*S. aureus* *S. pyogenes* *B. subtilis* *P. aeruginosa* *C. albicans*	Approximately 55.8%	In vitro cytotoxicity assay on cell line human skin fibroblast (HSF) show IC_50_ of 95.7% In vitro wound healing assay show monolayer of HSF were healed fast during 48 h and mostly reached to 90% for ZAg NCs	In vitro Inhib. zones: 20 mm 18 mm 21 mm 19 mm 17 mm	[165] (2022)
Cu-SER	Cu^2+^	Surface neutralisation MOF disintegration	*S. aureus* *E. coli*	-	Pro-osteogenic activity on human adipose-derived stem cells (hASCs).	In vitro Change in bacterial surface change, induction of morphological change.	[107] (2022)
ONP@ZnO_2_@ZIF-67(ONP@ZZ)	ROS	Chemodynamic therapy Photodynamic therapy	*MRSA* *E. coli*	-	In vitro cytotoxic and haemolysis assay on Murine L929 and raw 264.7 cells demonstrate biocompatibility. biosafe up to concentration of 25 µg mL^−1^. No haemolysis of red blood cells at concentration below 100 µg mL^−1^. No signs of inflammation or abnormality in vivo	In vitro The majority of MRSA was eradicated at concentration of 25 μg mL^− 1^ under irradiation of 660 nm light In vivo, increase in the average skin wound recovery to 98%	[110] (2022)

## 4. MOF and Polymer Hybrid Materials as Antimicrobial Materials

Looking at the medical applications of both MOFs and polymers, the promising potential of combining the two materials together can be easily realised. This is a topical area of research and multiple studies have been reported recently. Among the primary limitations of MOFs for their application is their crystalline powder form, which is difficult to integrate in purpose-built application devices. Integration of MOFs with polymer removes that barrier and makes it easier to customise and integrate in devices. In addition, polymers tend to have optoelectronic properties, and can also be flexible, and biocompatible. These desirable traits can be combined with MOFs to produce a hybrid multifunctional material [166]. Furthermore, MOF structures sometimes collapse when used for their desired applications, leading to a loss or decline in their efficacy over time. The incorporation of polymers aids in enhancing the stability of MOFs and thus alters their degradation rate, allowing for the early release of medical agents to be avoided, providing more control. The incorporation of polymers also helps to improve the behaviour of MOFs in water, making them more stable and dispersible, and enhance the capabilities of loading guest molecules. These effects are often induced by the physical and chemical properties of the polymer itself [48,167].

**Figure 17:**
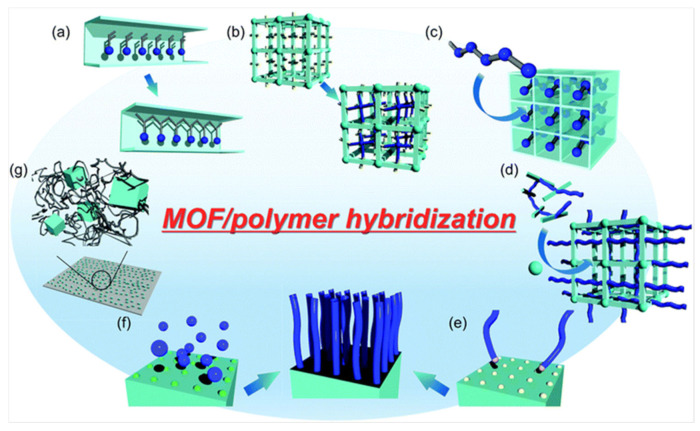
Different methodologies that can be used to synthesise MOF and polymer hybrids are shown. (**a**) Showing how the polymers can be made through polymerisations inside the nanochannels of MOFs. (**b**) Ligands being polymerised inside the pores of MOF. (**c**) Polymeric chains being introduced inside the nanochannels. (**d**) The polymers containing the ligands as their monomers. (**e**) Grafting polymer on MOFs and (**f**) grafting polymer from MOFs. (**g**) Mixed matrix membrane formation using polymer and MOFs. Reproduced with permission from [166].

There are multiple methodologies which can be used to synthesise these hybrid materials (Figure 17) [166]. For example, the polymers can be formed inside the nanochannels of MOFs through polymerizations, or polymeric chains can be introduced into them. Further examples of these synthetic approaches include the bonding of the MOF to the polymer that is or is not covalent, coordinating the polymers to the metal ions, or encapsulating the MOFs within the polymer [168].

The MOF–polymer hybrid materials have been found to have promising medical applications, such as in anticancer research. For example, one study utilised functionalised UiO-66 with polyethylene glycol chains by covalently attaching the MOF with the composite through mild bioconjugate reactions. The hybrid composite incorporated dichloroacetic acid inside the MOF pores and was pH responsive, allowing for the system to have enhanced selectivity and controlled delivery. In addition, the post-synthetic modification with PEG allowed to overcome the burst effect [169]. In addition to anticancer properties shown by multiple other studies, potential antimicrobial properties of these hybrid materials are also attracting attention [170,171]. Table 2 summarises the antimicrobial studies of recently studied polymer–MOF composites. 

**Figure 18:**
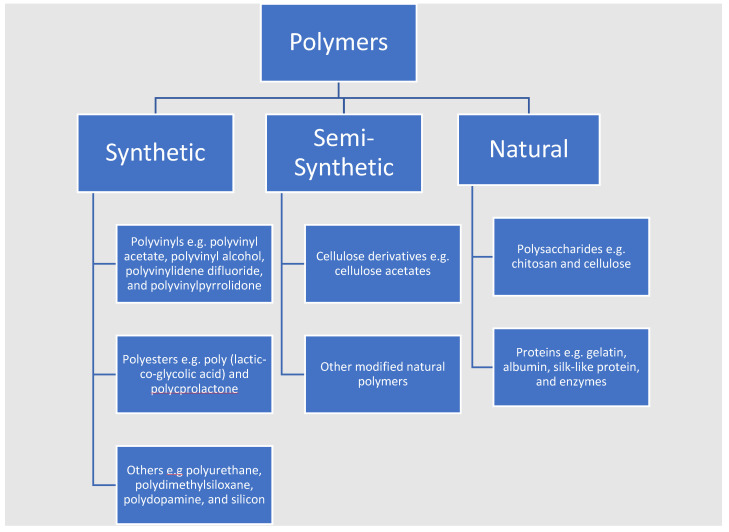
A scheme showing the synthetic, semi-synthetic, and natural polymers used for synthesising MOF–polymer composites for antimicrobial studies.

Research has investigated both the use of natural and synthetic polymers for developing polymer–MOF composites and their antimicrobial properties. Examples of both natural and synthetic polymers studied are summarised in Figure 18. Natural polymers consist of compounds which are found naturally and usually produced by organisms such as animals, plants, and microbes. These materials are biologically compatible and degradable, and they can also display their own biological activity. These compounds can be found with a range of individual chemical properties and structures which are difficult and sometimes not possible to re-create synthetically, they can also be adapted to different applications. Therefore, it is clear to see why natural polymers are promising in this research area. However, natural polymers do not tend to have the best mechanical qualities and have been observed to degrade rapidly when exposed to physiological conditions. This led to the development of synthetic polymers which can also be biologically compatible and degradable. These polymers can degrade via covalent bond hydrolysis, and the rate of degradation is dependent upon chemical factors such as steric hindrance at the cleavage site. The polymers can be synthetically adapted to suite the desired biological, physical, and mechanical properties making them highly suitable for biological applications [172]. With a focus on the recent trend of research in this area, several examples natural, synthetic and semi-synthetic types that have been used to make MOF and polymer hybrid materials as antimicrobial agents are discussed in the following section. 

**Table 2:** Summary of MOF–polymer composites for antimicrobial applications.

Polymer/MOF	Composition/Synthesis Method	Antimicrobial Agent	Applications	Mechanism	Target Organism	Antimicrobial Efficiency	Ref./Year
Cellulose–MOF199	Rapid solvent exchange upon dispersion in water	Cu^2+^	Water purification	MOF disintegration	*E. coli*	Optical density lower in the PolyMOF solution compared to controls after 4 h	[173] (2019)
Polylactic acid (PLA) fibres containing Co-SIM-1	2–6 weight % Co-SIM-1 to PLA. Electrospinning	Co^2+^	Membranes for biomedical applications	MOF disintegration	*P. putida* *S. aureus*	Inhib. zones: 23.6 ± 1.4 mm 25.4 ± 0.801 mm	[174] (2015)
PCL/Cur@ ZIF-8	0–35% MOF to PCL. Curcumin loaded during ZIF-8 synthesis, and solvent casting used to add PCL	Curcumin and ZIF-8 ROS Zn^2+^	Antimicrobial food packaging	Curcumin release ~doubled when Poly-MOF exposed to pH 5 compared to a neutral pH following 72-h	*E. coli* *S. aureus*	99.9% decrease in the growth of *E. coli* and *S. aureus* when over 15% of Cur@ZIF-8 was loaded. Detachment of bacteria	[175] (2019)
PCN−224 NPs@PCL	Up to 13.32 weight % PCN−224 NPs loaded Co-electrospinning	ROS/ photoirradiation	Antimicrobial wound dressing	Photoactivation	*S. aureus* *MRSA* *E. coli*	The survival rates of *S. aureus, MRSA,* and *E. coli* were 0.13%, 1.91%, and 2.06%, respectively.	[176] (2021)
MOF-525/PCL MMMs	10–30 weight % MOF-525 was loaded. Solvent casting	ROS/ photoirradiation	“Smart” biologically responsive material	Photoactivity	*E. coli*	Most colonies removed after 30 min up to 90 min of irradiation. Less than 80 viable colonies were left after 90 min or irradiation.	[177] (2017)
ZIF-8@PVA/CH/HA (polyvinyl alcohol, chitosan, hyaluronic acid)	0 to 1.0% wv of ZIF-8: composite Electrospinning	ROS/ photoirradiation	Biological materials for bone/tissue regeneration	Photoactivity	*B. cereus* *L. monocytogenes* *E. coli* *P. aeruginosa* *C. tropical* *C. glabrata* *C. albicans*	0.8%wv ZIF-8@PVA/CH/HA was the most active with the smallest inhibition zone being 9.67 ± 2.56 mm, and the largest 23.0 ± 2.0 mm.	[178] (2022)
I_2_@AuNR@SiO_2_@UiO-66 in (PVDF) film	8% and 25% of AuNR@SiO2@UiO-66: PVDF I_2_ content: 0.012 and 0.159 mg (mg film)^−1^ Drop casting	I_2_	Prophylactic treatment	Chemical effect NIR triggered release	*E. coli* *S. aureus*	Inhib. zone: 15.6 ± 3.8 and 41.6 ± 2.7 mm, for *E. coli* 19.5 ± 1.3 and 43.2 ± 4.3 mm, for *S. aureus*	[179] (2022)
UiO66@I_2_/PCL composite	0.5 and 1.0 wt% iodine Solvent casting	I_2_	Iodine-based antimicrobials	Chemical effect.	*S. aureus* *E. coli*	Inhib. zone: ~2 (between 3 and 5 mm) ~6 mm (between 11 and 12 mm)	[180] (2022)
MOF199@bamboo (carboxymethylated bamboo)	11.1 wt% Cu^2+^ two stage synthesis to immobilise MOF-199	Cu^2+^ MOF composite	MOF-coated wood-based materials	Physical disinfection Surface active metal sites	*E. coli*	Reduction in colony number by 38. 91.4% antibacterial ratio	[181] (2021)
PUF@Cu-BTC (Polyurethane foams)	Crosslinking reaction of castor oil and chitosan with toluene-2,4-diisocyanate.	CuBTC/ composite Active Cu^+2^ centres	Skin disease and wound treatment	Synergistic effect of composite and MOF	*P. aeruginosa* *K. pneumoniae* *MRSA*	97.8%, 99.9% and 77.6% reduction, respectively.	[106] (2021)
CP/CNF/ZIF-67 (Cellulose nanofibres, modified using sodium carboxylate groups)	20.5% MOF composition In situ synthesis	Co^2+^ 2-methylimidazole	Medical and health security	MOF disintegration	*E. coli*	Inhib. zone: 12 mm	[182] (2018)
ZIF-8/cotton fabrics (polydopamine templated cottons)	14.5% MOF/composite ratio in situ synthesis	Zn^2+^ -NH_2_ groups in polydopamine	Multifunctional textiles	MOF disintegration Formation of amine phosphate complexes	*E. coli*	Inhib. zone present. (not quantified)	[183] (2020)
Wool@MOF (HKUST-1 MOF)	in situ synthesis	Cu^2+^	Biologically functional fabrics	MOF disintegration	*E*. *coli* *S. aureus*	Before washing: 100% reduction after 24 and 48 h. After washing: 99.7% and 100% reduction for 24 and 48 h, respectively.	[184] (2019)
cotton@(ZIF-67)_3_/PDMS	12.97 wt% cobalt in situ synthesis, PSM with polydimethylsiloxane	Co^2+^	Multifunctional cotton fabric for use in the antibacterial and anti-ultraviolet field	MOF disintegration	*E. coli* *S. aureus*	Inhib. zone: 15 mm 15 mm (slight increase for *S. aureus*)	[185] (2021)
CS-Van-NMOFs	Vancomycin content: 9.87 ± 1.23% Mixing method	Vancomycin Metal ion	Antibiotic therapy of multiple drug resistant infections	Cargo release MOF disintegration	Vancomycin-sensitive *S. aureus* Vancomycin-resistant *S. aureus*	Refer to Table 3	[186] (2019)
PolyCu-MOF@AgNPs	Ag% wt: 7.24%; Cu% wt:3.46%	Cu^2+^ Ag^+^	Wound healing	MOF disintegration Cargo release	*E. coli* *S. aureus*	MIC: 10 µg mL^−1^ 10 µg mL^−1^	[187] (2022)
THY@PCN/PUL/PVA	Electrospinning	ROS/photoirradiation Thymol	Food packaging	Photodynamic therapy Cargo release	*E. coli* *S. aureus*	Inhibition of ~99% and ~98% for *S. aureus* and *E. coli*, upon irradiation, respectively	[188] (2021)
GelMA-graft-poly(AA-co-AAm)/MIL-53(Fe)/CS extract	Grafting	*Camellia sinensis* Fe^2+^	Antibacterial hydrogel wound dressing	(cargo release) (MOF disintegration)	*B. cereus* *S. aureus* *S. mutans* *K. pneumoniae* *P. aeruginosa C. albicans strain*	Inhib. zone: 27 ± 3 mm, 17 ± 4 mm, 23 ± 1 mm, 25 ± 2 mm, 20 ± 1 mm, 22 ± 4 mm, and 25 ± 3 mm, respectively	[189] (2022)
ZIF-8/cellulose	77.5% disposition ratio in situ synthesis	Zn^2+^	Composite filters	(MOF disintegration)	*E. coli*	Inhib. zone: 9.1 mm	[190] (2018)
MOF-199/cellulose	88.4% disposition ratio loading by in situ synthesis	Cu^2+^	Composite filters	(MOF disintegration)	*E. coli*	Inhib. zone: 15.2 mm	[190] (2018)
Ag-MOF/cellulose	87.2% disposition ratio Loading by in situ synthesis	Ag^+^	Composite filters	(MOF disintegration)	*E. coli*	Inhib. zone: 20.8 mm	[190] (2018)
Cu-BTC/cellulose	Surface grafting	Cu^2+^	Antimicrobial fabric	(MOF disintegration)	*E. coli*	MIC: 25 µM	[191] (2014)
CuBTC/silk	Layer by- layer	Cu^2+^ CuBTC	Antimicrobial fabric	(MOF disintegration)	*E. coli* *S. aureus*	Inhib. zone: 7.7–8.0 mm 6.5–7.5 mm	[192] (2012)
CuBTC/PVA	10 and 15% by weight Electrospinning	Cu^2+^ CuBTC	Antimicrobial fabrics	(MOF disintegration)	*E. coli* *S. aureus*	Inhib zone: *S. aureus* ranging from 2 to 4 mm	[193] (2018)
Cu_3_(NH_2_BTC)_2_ Cotton	Layer by layer	- Cu_3_(NH_2_BTC)_2_	Wound dressing	(Post-synthetic modification/MOF disintegration) (surface antibacterial properties, bacterial detachment)	*E. coli*	Reduction in viability of 4-log in modified MOF and 5-log in unmodified MOF, in 24 h	[194] (2018)
Cu-BTTri/chitosan	1%, 5% and 20% *w*/*w* mixing method	Cu^2+^	-	MOF disintegration Surface interaction	*P. aeruginosa*	Detachment of bacteria	[130] (2017)
CuBTC/polymer (nylon and polyester hybrid)	97.14–127.33 mg MOF (g fabric)^−1^ in situ synthesis	Cu^2+^ CuBTC	-	- (MOF disintegration)	*E. coli* *S. aureus* *C. albicans*	MIC: 60–64 mM 65–70 mM 62–67 mM	[195] (2018)
HKUST-1/chitosan	40% MOF: composite ratio from TGA freeze-drying	Cu^2+^ CuBTC	Wound dressing	(MOF disintegration) (contact-based action)	*E. coli* *S. aureus*	Shrinking of bacterial cell Upon 45 min of contact	[196] 2019
Ag NPs@ HKUST-1@ CFs (carboxymethylated fibres)	Deposition ratio: 31.64% by weight Ag wt%:4.79; Cu wt%: 13.3 in situ preparation	Ag^+^ Cu^2+^	Cellulose-based antibacterial materials (food and medical packaging)	Cargo release MOF disintegration	*S. aureus* *E. coli*	99.41% inhibition for S.aureus	[197] (2018)
2D Cu-TCPP(Fe)/GOx	2.5 ± 0.03 weight % glucose oxidase incorporated into MOF. Stirring and centrifugation	•OH	MOF-based nanozymes for biological applications	Glucose catalysis	*S. aureus* *E. coli*	Inactivation percentage of ~88~90%	[198] (2019)
MIL@GOx-MIL NR	7.5% glucose oxidase loaded Solvothermal method with centrifugation	•OH	MOF/enzyme hybrid nanoreactors	Glucose catalysis	Methicillin-resistant *staphylococcus aureus*	80 μg/mL MIL@GOx-MIL NRs antibacterial rate was greater than 99.99%.	[199] (2020)
MMNPs	Ultrasonication treatment followed by biomineralization process in alkaline conditions	ROS/photoirradiation	Antimicrobial photodynamic therapy	Photodynamic therapy	*S. aureus* *E. coli*	Following H_2_O_2_ addition and irradiation 99% *E. coli* and 90% *S. aureus* were eradicated.	[200] (2019)
PAN-PCN	0.1–0.6 wt% PCN-224 NPs in polyacrylonitrile Electrospinning	ROS/photoirradiation	To combat pathogen drug resistance and spreading	Photodynamic therapy	*S. aureus* *E. coli*	Antimicrobial photodynamic inactivation study (0.6 wt% PCN-224 NPs): *S. aureus—*4.70 log unit elimination *E. coli—*3.00 log unit elimination	[201] (2021)
TFC-Ag-MOF composites	In situ TFC functionalisation	Ag^+^	Antifouling membrane for FO applications	Ag^+^ release	*P. aeruginosa*	Bacterial mortality of 100% was nearly reached	[202] 2019

### 4.1. MOF and Synthetic Polymers as Hybrid Antimicrobial Materials (PolyMOFs)

**Figure 19:**
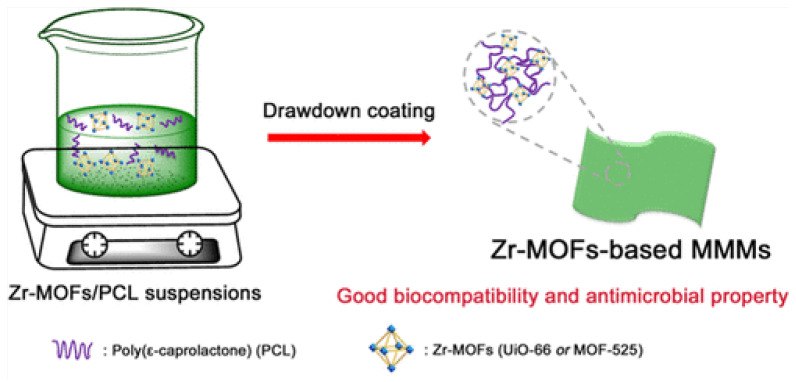
Scheme showing synthesis of mixed matrix membranes of poly(ε-caprolactone) and MOFs (UiO-66 and MOF-525). This scheme shows the synthesis method of Zr-MOF-based MMMs; a heated suspension of MOFs in PCL solution is then casted onto cover slides and left to dry, yielding antimicrobial MOF polymer composites. Reproduced with permission from [177].

Among the earliest studies that studied polymer–MOF mixed matrix membranes (MMM) for antimicrobial studies used poly(ε-caprolactone) (PCL) as a binder, due to its excellent biocompatible and biodegradable nature, and UiO-66 and MOF-525 as filler due to their good chemical stability, favourable biosafety, and a diverse range of functionalities (Figure 19) [177]. Irradiated MOF-525-based MMM generated ROS which acts as antimicrobial photodynamic agents and showed good efficiency against *E. coli*. 

In another study, a composite of UiO-66NH_2_ loaded with iodine (UiO66@I_2_) was mixed into a PCL matrix [180]. Iodine (0.5 and 1.0 weight %) was loaded into the composites. The loading of UiO66@I_2_ into the polymer did not affect its structure, although it did lead to a reduction in the static water angle. The composites with 0.5 and 1.0 weight % iodine showed inhibition zones of ~2 and 3–5 mm against *S. aureus*, respectively, and inhibition zones of ~6 and 11–12 mm against *E. coli*, respectively. Whereas when the polymer was loaded with iodine alone were tested, they displayed no antimicrobial effects. This difference in efficacy was because of adsorption and release of iodine by UiO-66NH_2_, in contrast to the polymer alone which had very little iodine content. This study found a method to trap iodine and create antimicrobial polymers.

In a study by Han et al. iodine release by a MOF composite was proposed as a new prophylactic treatment for bacterial infections [179]. Gold nanorods were covered with silica which were then encapsulated by UiO-66. This was done through a spray drying synthesis. The resultant microbeads were then incorporated into a polyvinylidene difluoride (PVDF) film with 8%, 25%, and 46% (wt%). Iodine was then loaded into the films with 0.012 mg_I2_ mg_film_^–1^ loaded into previously prepared the 8% film, 0.159 mg_I2_ mg_film_^–1^ loaded into the 25% film, and 0.434 mg_I2_ mg_film_^–1^ loaded into the 46% film. When the films were exposed to near infrared light the gold nanorods were irradiated and caused a photothermal effect, the heat produced led to the iodine being liberated. The iodine release measured can be seen in Table 2. The study also investigated the release of the of iodine using the 25% film with turning the near infrared light (52 or 224 mW cm^–2^) on for one minute and off for 3 min, and repeating this for 50 cycles. During the first cycle a burst effect was observed, with the iodine release plateauing once the 15^th^ cycle was reached, the release remained constant for the rest of the experiment. These release results only represent approximately 63% of the anticipated iodine release from the microbeads. This reduction in iodine release compared to the beads alone is due to the PVDF polymer acting as a diffusion blocker. The release data collected show that this system can be used for sustainable long release as well as being able to control by exposure to near-infrared light. For antimicrobial testing the films were irradiated at 224 mW cm^–2^ and 8% and 25% loaded films were tested against *E. coli*, showing inhibition zones of 15.6 ± 3.8 and 41.6 ± 2.7 mm, respectively. For the same study on *S. aureus* showed inhibition zones of 19.5 ± 1.3 and 43.2 ± 4.3 mm, respectively. The data indicate that this class of composite materials has promising potential for application as a controllable antimicrobial agent. 

**Figure 20:**
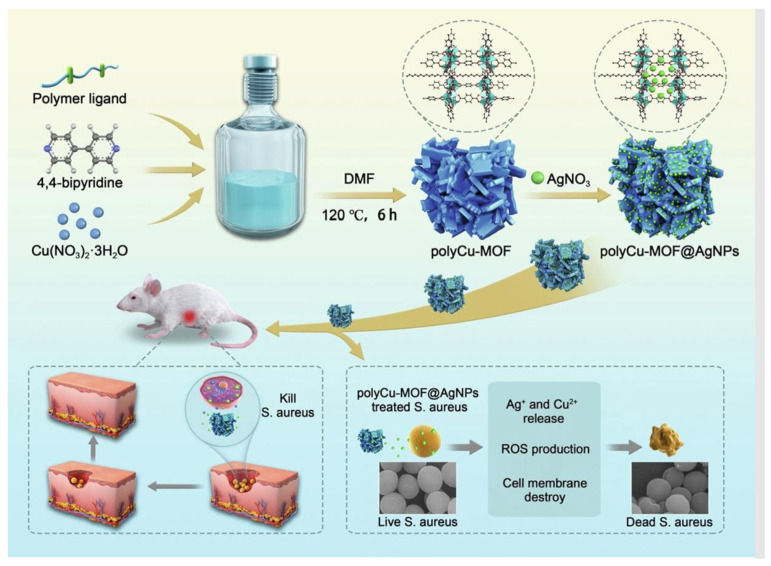
Scheme showing the synthesis of PolyCu-MOF@AgNPs and an illustration of the antibacterial properties of the product. The scheme shows in situ formation of loaded MOF/hybrid composites to create polyCu-MOF. The MOF composites are then post-synthetically treated with AgNO_3_ to yield polyCu-MOF@AgNPs. In vivo experiments on mice show improved healing of bacteria-infected skin wounds and allow for the regeneration of collagen and the healing of damaged tissues. SEM images of in vitro treated *S. aureus* showing the change of morphology of dead S. aureus and listing the antimicrobial mechanisms as Ag^+^ and Cu^2+^ release, ROS production as well as cell membrane destruction. Reproduced with permission from [187].

Another study developed a copper-based polymer MOF composite (PolyCu-MOF) to load silver nanoparticles and tested this as an antimicrobial to heal wounds (Figure 20) [187]. The PolyCu-MOF was synthesised with a polyether ligand that contained 1,4-benzenedicarboxylic acid, with 4,4′-bipyridine as a co-ligand, and copper as the metal coordination centre. The silver ions were then loaded using a wet chemical methodology to obtain PolyCu-MOF@AgNPs. In vitro cytotoxic and haemolytic effects of the composites were studied on mouse fibroblast cell line (L929) and human blood, respectively. MTT assay on L929 cells showed no toxic effects at concentrations between 2 and 20 µg mL^−1^. haemolysis tests showed that the haemolysis ratio depends on the PolyCu-MOF@AgNPs concentration. In addition, PolyCu-MOF was found to decrease the haemolytic effects of AgNPs by reducing the interaction with the cell membranes of red blood cells. The material consisted of thin nanosheet structures, an abundance of carboxyl moieties, structural defects, large surface area, and good porosity. The surface area of the MOF alone was 1121.7 m^2^g^−1^, which dropped to 598.15 m^2^g^−1^ when the polymer was incorporated. When the silver was loaded this dropped further to 45.39 m^2^g^−1^. The atomic % of silver and copper in the composite was 7.24% and 3.46%, respectively. This shows promising loading of the silver nanoparticles. In contrast to Cu-MOF@AgNPs, the PolyCu-MOF@AgNPs had a lower copper content and loaded more silver. This resulted in less copper ions but more silver ions being released, this enhanced the system’s biological compatibility, and caused a decrease in haemolysis. It was observed that after 48 h, 3.2 µg mL^−1^ copper and 3.08 µg mL^−1^ silver ions were released, respectively. The composite displayed antimicrobial activity through release of copper ions from the MOF and silver from the composite. This kills bacteria due to the cell integrity being damaged by reactive oxygen species being produced and the metabolism of the bacteria being disrupted. The minimum inhibitory concentration of the composite was found to be 10 μg mL^−1^ against *S. aureus* and *E. coli*. In vitro testing also showed that the product improved the healing efficiency of wounds which were infected with bacteria and promoted skin regeneration and dense collagen deposition. 

### 4.2. MOF and Semi-Synthetic Polymers Hybrid for Antimicrobial Studies

**Figure 21:**
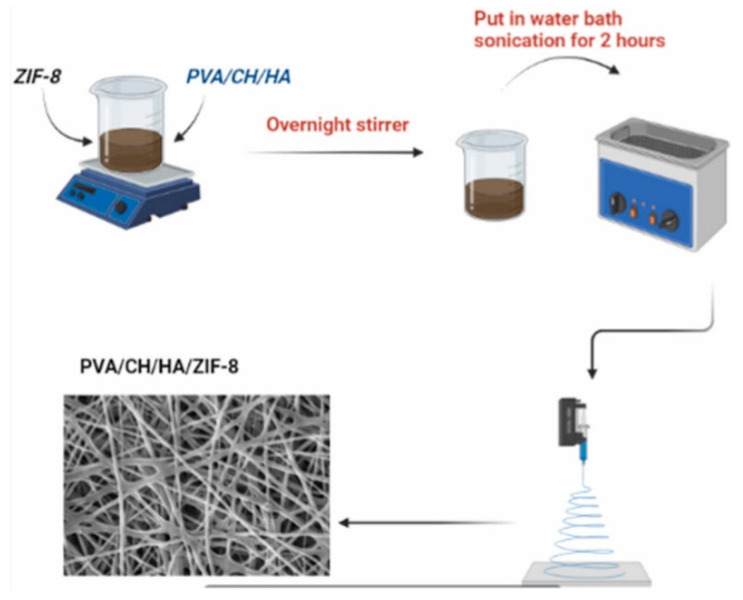
Scheme showing synthesis of ZIF-8@PVA/CH/HA composite. The synthesis of composites is done by stirring the ZIF-8 in PVA/CH/HA suspension overnight, then sonicating it for 2 h followed by electrospinning to yield fibres of ZIF-8@PVA/CH/HA. Reproduced with permission from [178].

A semi-synthetic polymer–MOF composite nanofibre (ZIF-8@PVA/CH/HA) (Figure 21) was developed by incorporating ZIF-8 nanoparticles into a mixture of polyvinyl alcohol (PVA), chitosan (CH), and hyaluronic acid (HA) (8:1:1 ratio) followed by electrospinning [178]. This allows for the integration of nanosystems with biologically compatible mats with potential application for regeneration of bones. Different loadings (weight/volume or *w*/*v*) of ZIF-8 were used to prepare the composite material and they were tested against a number of Gram-positive and -negative bacteria, as well as fungi species. The composite with 0.8% *w/v* of ZIF-8 showed highest efficacy. As suggested, this study provides a proof-of-concept for using biocompatible, antimicrobial MOF–polymer composites in applications such as bone regeneration and tissue grafting. The polymer composites would act similar to a tissue-like structure that can be implanted for bone regeneration, due to its osteogenic and osteoinductive properties [203]. 

**Figure 22:**
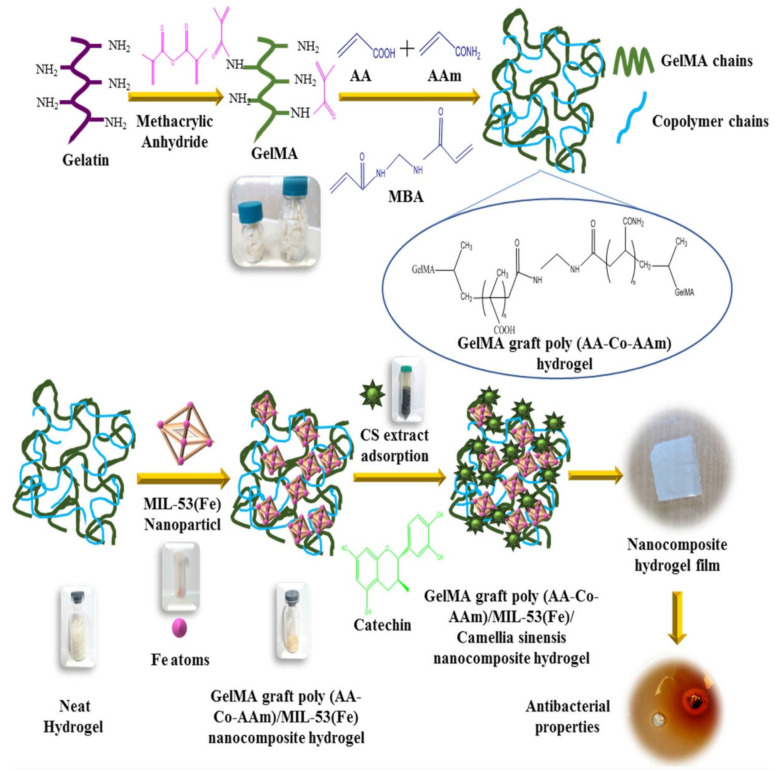
The scheme showing the synthesis of (GelMA-graft-poly(AA-co-AAm)/MIL-53(Fe)/CS extract). Top shows the preparation steps of GeIMA and copolymer chains, then, MIL-53(Fe) MOF is then incorporated to form the MOF–polymer composite. The composite is post-synthetically loaded with CS extract. The scheme shows pristine hydrogel, prior to adding MOFs to yield the nanocomposite. the nanocomposite was studied in vitro for its antimicrobial effect. Reproduced with permission from [189].

A hydrogel composite consisting of modified gelatine and an iron-based MOF was developed by Hezari et al. for use as an antimicrobial bandage (Figure 22) [189]. Methacrylate anhydride was used to modify gelatine, a biologically compatible naturally occurring polymer, to create gelatine methacrylate. Aqueous polymerization was then used to graft acrylic acid and acrylamide onto the product. The MOF was synthesised using a solvothermal method and mixed into the hydrogel composite. The MOF was incorporated to add porosity, strengthen the product, improve the ability of the gel to carry antimicrobials, and limit the toxicity of the product. TGA analysis carried out showed how the MOF improved the thermal stability of the hydrogel. The MOF improved porosity and as a result increased the ability of the composite to absorb water compared to the hydrogel alone. It was observed that the water absorption capacity for the composite hydrogel was 547.96 g/g, whereas it was 400.10 g/g for the gel alone, and it was observed that the products’ ability to absorb water was affected by the pH*. Camellia sinensis*, a herbal antimicrobial agent, was then loaded into the composite to obtain the final product (GelMA-graft-poly(AA-co-AAm)/MIL-53(Fe)/CS extract) with 6.25 mg/mL, 12.5 mg/mL, and 25 mg/mL of camellia sinensis into the hydrogels. The inhabitation zone size was measured when the composite was tested against *B. cereus*, *S. aureus*, *S. mutans*, *E. coli*, *K. pneumoniae*, *P. aeruginosa*, and *C. albicans* strain, with zones measuring 27 ± 3 mm, 17 ± 4 mm, 23 ± 1 mm, 25 ± 2 mm, 20 ± 1 mm, 22 ± 4 mm, and 25 ± 3 mm, respectively. The results of this research point to this hydrogel composite being a promising product to be incorporated into an antimicrobial bandage. 

In another study, cellulose acetate, a semi-synthetic polymer, derived from natural cellulose, was used [173]. In this study, the polymer was incorporated with HKUST-1 (MOF-199) for applications in water filtration and remediation. Imaging was carried out on the cellulose alone and it revealed the presence of macropores. The images of the PolyMOF also showed that the MOFs when loaded had aggregated in these pores, with the core of the product appearing to be mainly hollow. Release studies showed that whilst the MOF alone completely degrades after approximately four hours, once combined with the polymer decomposition was slowed down. The HKUST-1 alone displayed a high copper ion loss after half an hour, whereas the PolyMOF showed three times less copper ion released. This shows that cellulose improves the stability of the MOF. The antimicrobial activity of the composite was tested as it was found that when a bacterial cell touches the surface of PolyMOF it results in a toxic amount of copper ions diffusing inside the cell wall. In this composite HKUST-1 itself acts as the antimicrobial agent, by releasing these copper ions. This activity was measured by *E. coli* being used to inoculate a solution of tryptic soy broth and incubated at 37 °C. The optical density was then obtained at certain time points, and it was found that after 4 h this was lower in the PolyMOF solution compared to the controls, suggesting antimicrobial properties of the composite.

**Figure 23:**
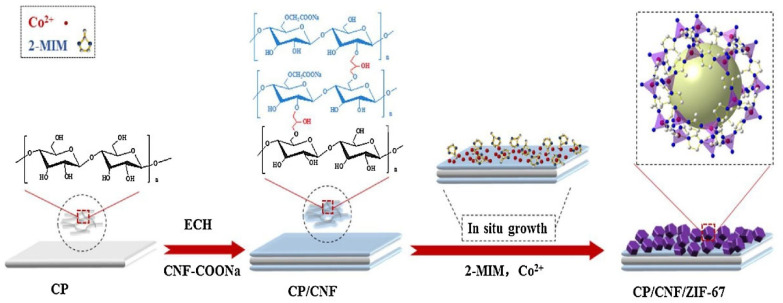
The scheme showing the synthesis of CP/CNF/ZIF-67 composite. The fabrication process is done by first grafting CNF on the surface of CP via Williamson reaction, then ZIF-67 is reacted on situ, to yield CP/CNF/ZIF-67. Reproduced with permission from [182].

In another study, Rahimi et al. reported a semi-synthetic polymer–MOF composite for antimicrobial applications (Figure 23) [182]. Cellulose nanofibres (CNF) were modified using sodium carboxylate groups and grafted upon the surface of cellulose papers (CP) using epichlorohydrin as a crosslinker. ZIF-67 was then synthesised in situ on the cellulose composite to obtain CP/CNF/ZIF-67. It was found that the incorporation of 2.5 weight % cellulose nanofibres resulted in a MOF loading ratio of 20.5%. Through tensile testing it was observed that the composites with 2.5 weight % carboxylated cellulose nanofibre showed brilliant mechanical abilities when exposed to dry and damp environments. CP/CNF/ZIF-67 displays antimicrobial activity by the release of Co^2+^ ions from ZIF-67, which results in a change to the pathogens environment causing an ion imbalance and the ion channels being broken. The MOF also releases 2-methylimidazole, which bonds with ions (Ca^2+^ and Mg^2+^) within the cells and results into breaking of the cell walls leading to cell death. Antimicrobial studies were carried out on *E. coli* and the inhibition zone was found to be 12 mm in diameter. 

### 4.3. MOF and Natural Polymers as Hybrid Antimicrobial Materials

**Figure 24:**
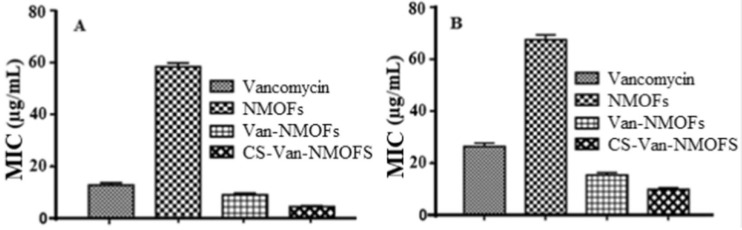
Minimum inhibitory concentration of vancomycin, Van-NMOFs, NMOFs and CS-Van-NMOFs. Graph (**A**) shows when they were tested against sensitive *S. aureus*. Graph (**B**) shows when they were tested against resistant *S. aureus*. Reproduced with permission from [186].

Chitosan is a natural polymer that has been used widely in developing hybrid biocompatible composites. A study by Ghaffar et al. reported a composite of chitosan and MOF (MIL-153) pre-loaded with vancomycin [186]. The pre-loaded MOFs were mixed with chitosan to form a homogenous solution, and the final product (CS-Van-NMOFs) was obtained. Chitosan, a deacetylated product from chitin, has bio-adhesive properties as they can interact with negatively charged bacteria cells surface, without bonding to them. This allowed for an effective contact between the MOF and bacterial cells, which also lead to more vancomycin uptake by bacterial cells. The MOF itself also releases metal ions which produce reactive oxygen species when internalised by bacteria. 11.49 ± 2.65% vancomycin was loaded into the MOF alone. Once encapsulated in chitosan, this dropped to 9.87 ± 1.23%, and was likely due to drug molecules that were loose bound to the surface of the MOF being displaced or removed. Surface area analysis showed the average pore size of the MOF dropped from 9.671 to 9.135 nm after drug loading, and then 6.671 nm after chitosan incorporation. The same trends for BET surface area (996.17 m^2^g^−1^ > 22.92 m^2^g^−1^ > 14.14 m^2^g^−1^) was observed. Both vancomycin-sensitive and -resistant *S. aureus* were tested against the PolyMOF. The results showed that the antimicrobial ability of the drug was increased once incorporated into chitosan encapsulated MIL-53 and led to an increase in bactericidal activity against both bacterial species (Figure 24 and Table 3). 

**Table 3:** Minimum inhibitory concentration, minimum bactericidal concentration, and concentration causing inhibition of 50% bacteria of CS-Van-NMOFs when they were tested against sensitive *S. aureus* and resistant *S. aureus* [186].

Bacterial Species	Minimum Inhibitory Concentration (μg/mL)	Minimum Bactericidal Concentration (μg/mL)	Concentration Causing Inhibition of 50% Bacteria (μg/mL)
Vancomycin-sensitive *S. aureus*	3.81 ± 1.13	127.81 ± 2.66	16.73 ± 0.88
Vancomycin resistant *S. aureus*	8.92 ± 0.69	169.34 ± 2.58	24.06 ± 1.18

AFM showed that the bacterial strains morphology had been completely distorted after being treated with the CS-Van-NMOFs. The results of this study showed that this system could be used as an alternative treatment for vancomycin resistant bacteria revealing a promising potential treatment for multidrug resistant bacterial infections.

In another study, MOFs were incorporated into cellulose for air purification and also studied for their antimicrobial properties [190]. The composites were synthesised by first forming a suspension of the cellulose fibres (CF), then stirring at room temperature, and adding the reagents to synthesise the MOFs. The resultant solutions were then heated in an oven in an autoclave, followed by washing with water and ethanol and then the final products, ZIF-8@CF, MOF-199@CF, and Ag-MOFs@CF, were freeze dried. The MOFs were loaded by two different mechanisms. For ZIF-8@CF, Zn^2+^ was adsorbed onto cellulose surfaces through electrostatic interactions. The linkers were then added to form ZIF-8. The ions also interact with hydroxyl groups on the fibres via hydrogen bonding. Whereas for both MOF-199@CF and Ag-MOFs@CF the linkers first form ester bonds with the hydroxyl groups on the surface of cellulose, with the metal ion then interacting with them and forming the MOF in situ. The deposition ratios were found to be 77.7% for ZIF-8@CF, 88.4% for MOF-199@CF and 87.2% for Ag-MOFs@CF. It was found that in ZIF-8@CF, there was a 19.75 weight % of zinc; in MOF-199@CF, there was a 14.12 weight % of copper; and in Ag-MOFs@CF, there was a 37.04 weight % of silver present. The products showed antimicrobial activity by releasing metal ions and organic linkers from the MOF structures, which lead to the damage of cell membranes and to the DNA becoming fragmented. *E. coli* was tested against the Poly-MOFs through measuring inhibition zones. All the Poly-MOFs and MOFs individually showed antibacterial activity, through measuremets of zones of inhibition. However, it was found that Ag-MOFs@CF (20.8 mm) had the highest level of antibacterial activity followed by MOF-199@CF (15.2 mm), with ZIF-8@CF (9.1 mm) showing the least amount of activity with the smallest inhibition zones. The same pattern was observed for the MOFs alone, although they showed a higher level of activity compared to their Poly-MOFs.

In another study, cotton fabrics were used with polydopamine and incorporated ZIF-8 to develop an antimicrobial textile [183]. The MOF was synthesised using an in situ solvothermal synthesis upon the polydopamine templated cottons surface. It was found that the calculated mass fraction of the MOF incorporated was 14.5%, showing the MOF had been successfully incorporated. The MOF and the polydopamine in the fabric presented antimicrobial activity. The Zn^2+^ ions released from the MOF inhibit bacterial growth through breaching the cells. The NH_2_ groups in polydopamine can interact with phosphates in the pathogenic cells leading to the formation of amine-phosphate complexes, resulting in cell damage. Sizeable inhibition zones were observed when the product was tested against *E. coli*. 

In another, study bamboo was used and functionalised using MOF199 (HKUST-1) for antimicrobials applications [181]. Bamboo was pre-treated using delignification and carboxymethylation, following this, a green two staged synthesis was then carried out to immobilise the MOF on the bamboo’s surface. The resultant product (MOF199@bamboo) consisted of well-distributed MOFs with good adhesion on the surface of carboxymethylated bamboo. It was found that 11.1% copper had been incorporated in the MOF199/delignified and carboxymethylated bamboo product. The antimicrobial activity for this product is caused by the MOF itself, they act through their surface-active metal sites and metal ions, and the bacteria also interact with the MOFs physically. All these factors work together to destroy the bacterial cells. It was found that the composite made on delignified and carboxymethylated bamboo showed the highest level of antimicrobial activity against *E. coli*. The results indicate that the product has promising properties for applications in the design of MOF-coated wood-based products. 

## 5. MOF and other Miscellaneous Agents as Hybrid Antimicrobial Materials

**Figure 25:**
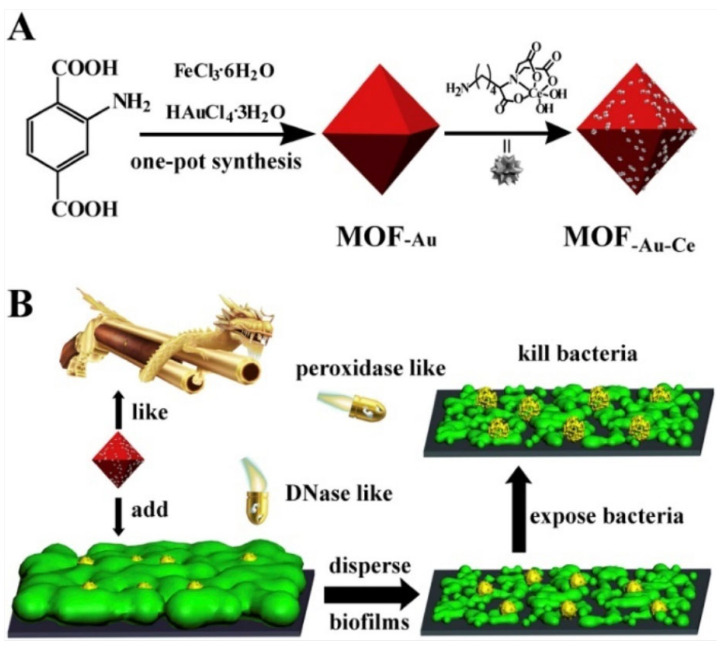
(**A**) Reaction scheme showing the synthesis of MOF_Au-Ce_, and the post-synthetic treatment of attaching Ce to MOF surface. (**B**) Antimicrobial activity of MOF_Au-Ce_ is illustrated showing that the MOFs can have dual enzyme mimetic activity, peroxidase like, and DNase like. Reproduced with permission from [204].

Aside from polymers, a range of other materials have been studied for incorporation with MOFs to produce novel antimicrobial agents—for example, a MOF (Au-doped MIL-88B) with a cerium-based nanozyme studied for its antimicrobial properties (Figure 25) [204]. Biofilms contain extracellular polymeric substances, extracellular DNA and bacterial cells together. This makes it difficult to permeate and cause bacterial death. This material was developed to mimic the activity of deoxyribonuclease and peroxidase to act against in vivo biofilm formation. The nanozyme acts by being able to hydrolyse extracellular DNA and cause disruption to biofilms. Meanwhile, the MOF displays activity similar to that of peroxidase, which can eliminate bacterial cells which were exposed in biofilms when hydrogen peroxide is present. This inhibits bacteria from recolonising and thus forming biofilms. The MOFs were synthesised using a solvothermal method, using different concentrations of gold (III) chloride trihydrate to produce MOF_−4.9Au_, MOF_−2.5Au_ and MOF_−1.6Au_. A solvent-based synthesis was then used to incorporate the cerium complex, this led to the formation of the final product MOF_−2.5Au-Ce_. With two forms of nanozyme being used, it greatly enhances the efficacy of the material to combat biofilm formation. Using this product avoids the use of naturally occurring enzymes that tend to be expensive and vulnerable. MOF_−2.5Au-Ce_ was used alongside H_2_O_2_ to treat subcutaneous abscesses caused by *S. aureus* in mice. To check how the infected sites were healing, an excision was taken of the tissue to count the number of surviving bacterial colonies. It was found that only ~50 × 10^7^ CFU/mL bacteria had survived the treatment, showing that the product had significantly reduced the number of inflammatory cells and aided in healing the wounds. This study produced a promising material for antimicrobial applications. 

Another study developed a different strategy for the inhibition of biofilm formation by using a porphyrin-based MOF (PCN-224) which was decorated with CeO_2_ [205]. The MOF acts by generating ROS which can kill planktonic bacterial cells. CeO_2_ acts by inhibiting extracellular adenosine triphosphate, which is essential in bacteria’s ability to adhere to surfaces and to form biofilms, which leads to the bacterial cells being inhibited from adhering to the surface. It was found that 30 weight % cerium was successfully incorporated into the MOF. The system was tested for its ability to inhibit extracellular adenosine triphosphate, it was found that after 12 h less than 40% of the extracellular adenosine triphosphate was left. It was then determined that the presence of CeO_2_ did not alter the photodynamic efficiency of the MOF. The compound was then tested against *S. aureus* biofilm formation. The product was tested at different concentrations and being irradiated at 638 nm for 5 min. It was found that 50 µg mL^−1^ of the MOF was able to reduce the biomass by >70%, and when the concentration went up to 50 µg mL^−1^ this increased to 90% of the biomass being inhibited from forming. The samples were then tested against subcutaneous abscesses in Kunming mice which was induced by the subcutaneous injection of *S. aureus* solution, and after 5 days there were no longer any evidence of an abscess. The compound was also examined for its cytotoxicity and biological compatibility on human embryonic kidney HEK 293T cells via standard methyl thiazolyl tetrazolium (MTT) assay, and it showed insignificant toxicity and promising biocompatibility. Results indicated that this product may be suitable for potential applications as an antimicrobial agent for inhibiting biofilm formations. 

**Figure 26:**
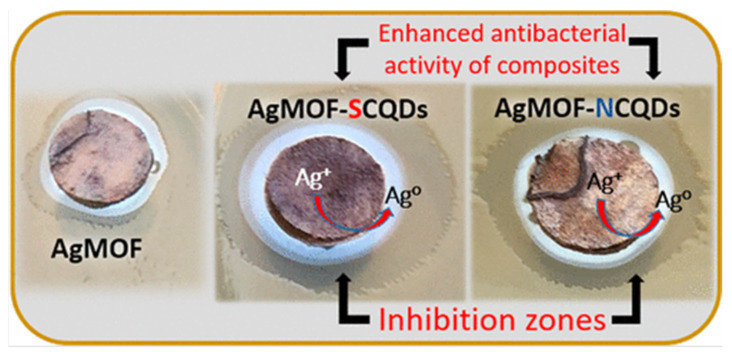
Antimicrobial activity of AgMOFs-S1 and AgMOF-N1 are illustrated with the inhibition zones around the composites. The Ag^+^ released from MOFs have high affinity to the S-compounds of cells, hence forming Ag° and Ag_2_S which are important for the antimicrobial action. Reproduced with permission from [206].

In a separate study, sulphur- and nitrogen-carbon quantum dots have also been incorporated with MOFs consisting of Ag and 1,3,5-benzenetricarboxylic acid, to make potential antimicrobial agents (Figure 26) [206]. The quantum dots were made using a hydrothermal method, an aqueous-based method was used to incorporate the MOFs and produce the AgMOFs-S1 and AgMOF-N1. The MOF composites displayed antimicrobial activity through a synergistic method of activity which led to an improvement in efficiency compared to the MOFs and carbon quantum dots alone. A transfer in charge between the MOF and carbon quantum dots allowed for an electrostatic interaction to occur between the composite and bacteria’s cell membrane. An increase in antimicrobial activity was connected to the nanorod morphology and certain aspects of its surface chemistry. The degradation of the AgMOF within the extracellular environment caused silver ions to be released. These ions have a large affinity towards sulphur compounds from the cell’s physiology. The metallic silver and silver sulphides formed were thought to be primarily responsible for the composites ability to stop bacterial cell growth. It was found that for AgMOFs-S1 17.1% and AgMOF-N1 17.4% silver was incorporated into the composites. The composites were tested for cytotoxicity and found not to display toxicity towards living cells. Antibacterial properties of the composites were studied against *B. subtilis* and *E. coli*. A minimum inhibitory concentration of 4 μg mL^-1^ for AgMOF-N1 and 8 μg/mL for AgMOF-S1 were found for *E. coli*, and 32 μg mL^-1^ for AgMOF-N1 for *B. subtilis*, with AgMOF-S1 showing less antimicrobial ability than the MOF alone, which had a minimum inhibitory concentration of 16 μg mL^-1^. The inhibition zones observed for *E. coli* were on average 12.9 ± 0.3 mm for AgMOF-S1 and 14.4 ± 0.7 mm for AgMOF-N1. Smaller inhibition zones were observed against *B. subtilis*, with size of 11.9 ± 0.7 mm on average for AgMOF-S1 and 12.3 ± 0.8 mm on average for AgMOF-N1. 

## 6. Use of Computational Modelling in Drug Delivery Studies Using MOFs

Although earlier studies on MOFs for drug delivery applications are focused on experimental investigation, a more recent trend can be observed on using computational modelling and simulation techniques as tools to predict optimum interactions between the MOFs and drug molecules. For example, quantum mechanical calculations were employed to accurately predict the highest loading capacity of gemcitabine-loaded IRMOF-74-III reaching as high as 95 wt%, surpassing other drug delivery agents, such as lipid-coated silica [207]. DFT calculations were used to find the most stable conformation of gemcitabine inside MOF pores, and role of functional groups of MOF linker on drug adsorption capacity by showing that the most significant interaction between the hydroxyl group of the linker and the carboxylate group of gemcitabine. In another study, molecular simulations helped in predicting the diffusion and adsorption patterns of MOF-74 MOF materials as a binary drug carrier for 5-flourouracil and methotrexate, calculating the highest loading capacities of 2.8 and 4.2 g g^−1^ for methotrexate and 5-flourouracil, respectively [208]. Another study has investigated the effect of the size of different s-nitrosothiols on the release behaviour of the antimicrobial NO from Cu-based MOFs, based on condensed phase classical molecular dynamics simulation [209]. In silico, many factors that contribute to loading capacity, release behaviour and drug–MOF interactions can be studied effectively [210]. The development of computational techniques can dramatically shorten the time needed for experimental investigations—for example, drug loading simulations predicted ZIF-8 to have the highest loading capacities compared to ZIF-90 and ZIF-67 [211], allowing to bypass the time taken to optimise by trial-and-error method, and hence rapidly minimise the time and resources needed to perform MOF loading studies. The synergy between computational and experimental studies in the field of drug delivery using MOFs can provide us with greater insight into interactions between drugs and host materials for optimised delivery. In addition, it can provide us with a greener approach of material investigations and fast-forward the time involved in experimental research by narrowing down the best fit for a combination of MOFs and drug molecules.

## 7. Conclusions and Outlook

The development of antimicrobial agents revolutionised medicine, by improving clinical outcomes of infections that were once thought to be untreatable and fatal. However, the excessive use of such agents, including antibiotics, antifungal, and antiviral drugs, has resulted in AMR. Although several generations of these agents were developed to combat this issue, AMR has become a major concern and is now among the biggest medical challenges globally. In this review, an overview of alternative methods of combating AMR using MOFs has been provided. MOFs are a highly versatile class of crystalline materials, made up of three-dimensional networks of metal clusters and polytopic organic ligands. Suggested mechanisms of how MOFs prevent microbial growth have been discussed. MOFs can be used as drug delivery vehicles, owing to their porous structure, and their ability to slowly release loaded chemicals. MOFs can also act as a reservoir for the slow release of antimicrobial metal ions, such as Cu^2+^, Zn^2+^and Ag^+^ and antimicrobial linkers, as a result of their slow degradation and diffusion. Studies also covered the photoactive antimicrobial action of MOFs, through the incorporation of appropriate photo-responsive units into MOFs, either by post-synthetic modifications or intrinsically within the structure of the MOFs. Additionally, other antimicrobial mechanisms of MOFs were also explored, such as chelation, enzyme-mimetic action via Fenton-like reactions, physical disinfection, chemodynamic therapy (CDT), neutralisation, and ultrasound-mediated killing, via sonodynamic therapy (SDT). The antimicrobial efficacy of the majority of the MOFs and their hybrid materials reported in this review have been investigated against *E. coli*, *S. aureus*, *K. pneumoniae, S. pneumoniae, A. baumannii,* and *P. aeruginosa**,*** as a list of leading pathogens linked to AMR. Efficacy studies have demonstrated enhanced antimicrobial action, often resulting in lowered MIC, when an antimicrobial agent is combined with a MOF. Additionally, the use of MOFs has demonstrated better in vivo and in vitro outcomes in several studies compared to the commonly used antimicrobial agents when used alone, as a result of their additive and synergistic antimicrobial action. It was also shown that multiple antimicrobial mechanisms can be simultaneously present. A brief overview of recent studies on incorporation of MOFs into polymer composites and their antibacterial studies have also been provided. The integration of MOFs within a composite explores the potential of this promising class of materials for real-life biomedical applications, by enhancing its physical properties, drug-release rates, overcoming its limitations due to granular form, and resulting into a uniform, application-appropriate dosage formulations. More recently, computational modelling is also being used as a predictive tool for drug–MOF interactions, and it is expected that the use of modelling will increase rapidly in the coming years. However, being an emerging area, there are several aspects that need more studies which will help to understand this class of materials for their biomedical applications for antimicrobial properties. MOFs have demonstrated their potential as an efficient drug delivery system, and in vitro biocompatibility studies on mice and human cells showed promising results. In vivo studies showed their ability for intestinal penetration [212], and enhancement of drug oral bioavailability [213]. In addition, several studies investigated the formulation of MOFs into inhaled [214], ophthalmic [215], and topical dosage forms [216]. As a future direction of research in this area, more emphasis is needed to understand the biocompatibility of these hybrid materials [217]. More research is also required to understand the drug delivery mechanism, biodistribution and interactions with human cells and physiology, in order to move this exciting class of materials closer to the clinic.

## Data Availability

Data is contained within the article and cited references.

## Abbreviations

AA: Acrylic acid; AFM: Atomic force microscopy; AMP: Antimicrobial peptides; AMR: Antimicrobial resistance; AzA: Azelaic acid; BET: Brunauer–Emmett–Teller; BioMOF: Bioactive metal–organic framework; bipy: 2,2′-Bipyridine; BIT: Beijing Institute of Technology; BTC: 1,3,5-benzenetricarboxylic acid; BTTri: 1,3,5-tris(1H-1,2,3-triazol-5-yl)benzene); CD: Cyclodextrin; CDT: Chemodynamic therapy; CF: Cellulose fibres; CFU: Colony forming units; CH: Chitosan; CIP: Ciprofloxacin; CNF: Cellulose nanofibres; CP: Cellulose papers; CS: *Camellia sinensis*; Cur: Curcumin; DNA: Deoxyribonucleic acid; FO: Forward osmosis; GelMA: Gelatine methacrylate; GOx: Glucose oxidase; H_3_BTB: 1,3,5-tris(4-carboxy-phenyl)benzene; HA: Hyaluronic acid; Hbim: Benzimdazole; HKUST: Hong Kong University of Science and Technology; Hmim: 2-methylimidazole; HMME: hematoporphyrin monomethyl ether; ISC/SUF: Iron-sulphur cluster/Sulphur formation; LPS: Lipopolysaccharides; MB: Methylene blue; MBC: Minimum bactericidal concentration; MIC: Minimum inhibitory concentration; MIL: Materials Institute Lavoisier; MMMs: Mixed-matrix membranes; MMNP: Multicomponent nanoplatform; MOF: Metalorganic framework; MRSA: Methicillin-resistant *Staphylococcus aureus*; MRSE: Methicillin-Resistant *Staphylococcus epidermidis*; NAP: 4-sulfo-1,8-naphthalimide; NIR: Near infrared radiation; NP: nanoparticles; NR: Nanoreactors; ONP: Organic NIR probe; PAN: Polyacrylonitrile; PCL: poly(ε-caprolactone); PCN: Porous coordination network; PDMS: polydimethylsiloxane; PEI: Polyethyleneimine; PLA: Polyacetic acid; PSM: Post-synthetic modifications; PTA=O: 1,3,5-Triaza-7-Phosphaadamantane-7-Oxide; PUF: Polyurethane foams**;** PUL: Pullulan; PVA: Polyvinyl alcohol; PVDF: Polyvinylidine fluoride; PYDC: pyridine-3, 5-dicarboxylic acid; ROS: Reactive oxygen species; SBU: Secondary building unit; SDT: Sonodynamic therapy; SEM: Scanning electron microscopy; SER: Sericin; NC: Nanocages; TCPP: Tris (1-chloro-2-propyl) phosphate; TDM: tetrakis [(3,5-dicarboxyphenyl)-oxamethyl] methane; TFC: Thin-flim composite; TGA: Thermogravimetric analysis; THY: Thymol; UiO: Universitetet i Oslo; UoB: University of Birjand; US: Ultrasound; Van: Vancomycin; ZAG: ZIF8/Au-GOx NPs; ZIF: Zeolitic imidazolate framework; ZPM: zirconium-based porphyrinic MOF; ZZ: ZIF-67-shell-coated ZnO_2_.
